# Supplementary material for: Ximaoornatins A–C, Polyoxygenated Diterpenoids from the Hainan Soft Coral Sinularia ornata
Source: Mar Drugs. 2022 Mar 20;20(3):218. doi: 10.3390/md20030218 (PMC8950289; doi:10.3390/md20030218)
Supplement: Supplementary file 1 [file marinedrugs-20-00218-s001.zip › marinedrugs-1634679-supplementary.pdf]

Supporting Information

Ximaoornatins A–C, Polyoxygenated Diterpenoids from the Hainan Soft Coral *Sinularia ornata*

Li-Li Sun [1,2], Xu-Wen Li [1,2,3,*] and Yue-Wei Guo [1,2,3,*]

[1] State Key Laboratory of Drug Research, Shanghai Institute of Materia Medica, Chinese Academy of Sciences, 555 Zu Chong Zhi Road, Zhangjiang Hi-Tech Park, Shanghai 201203, China; sunlili202105@126.com (L.-L.S.)

[2] University of Chinese Academy of Sciences, No. 19A Yuquan Road, Beijing 100049, China

[3] Drug Discovery Shandong Laboratory, Bohai Rim Advanced Research Institute for Drug Discovery, Yantai 264117, China

* Correspondence: xwli@simm.ac.cn (X.-W.L.); ywguo@simm.ac.cn (Y.-W.G.); Tel.: +86-21-50805813 (Y.-W.G.)

Contents

1. Experimental Section 3

    1.1 General experimental procedures 3

    1.2 Biological materials 3

    1.3 Extraction and isolation 3

2. Original data for compounds **1–5** 5

    2.1 X-ray crystallographic analyses of **1** 5

    2.2 NMR, MS, and IR spectra of compounds **1−5** 7

1. Experimental Section

1.1 General experimental procedures

IR spectra was recorded on a Nicolet 6700 spectrometer (Thermo Scientific, Waltham, MA, USA); peaks are reported in cm^{-1}. Melting points were measured on an X-4 digital micro-melting point apparatus. Optical rotations were measured on a Perkin-Elmer 241MC polarimeter (PerkinElmer, Fremont, CA, USA). The NMR spectra were measured at 300 K on DRX 500 and Avance 600 MHz NMR spectrometers (Bruker Biospin AG, Fallanden, Germany); Chemical shifts are reported in parts per million (δ) in $CDCl_3$ (δ_H reported referred to $CHCl_3$ at 7.26 ppm; δ_C reported referred to $CDCl_3$ at 77.16 ppm) and coupling constants (J) in Hz; assignments were supported by ^1H–^1H COSY, HSQC, HMBC, and NOESY experiments. HR-ESIMS was carried out on a Waters Q-TOF Ultima mass spectrometer (Waters, MA, USA). HREIMS spectra was carried out on a Thermo DFS mass spectrometer. Semi-preparative HPLC was performed on an Agilent-1260 system equipped with a DAD G1315D detector using ODS-HG-5 (250 mm × 9.4 mm, 5 μm) by eluting with CH_3OH–H_2O or CH_3CN–H_2O system at 3.0 mL/min. Commercial silica gel (200−300 and 300−400 mesh; Qingdao, China) was used for column chromatography (CC). Precoated Si gel plates (Merck Chemicals (Shanghai) Co., Ltd, G60 F254) were used for analytical TLC. Sephadex LH-20 (Amersham Biosciences) was also used for CC. All solvents used for column chromatography and HPLC were of analytical grade (Shanghai Chemical Reagents Co., Ltd.) and chromatographic grade (Dikma Technologies Inc.), respectively. X-ray diffraction studies were carried out on a Bruker D8 Venture diffractometer with Cu Kα radiation (λ =1.54178 Å).

1.2 Biological materials

Specimens of the soft coral *Sinularia ornata*, identified by Prof. Xiu-Bao Li from Hainan university, were collected along the coast of Ximao Island, Hainan province, China, in 2018, and were frozen immediately after collection. A voucher specimen (18-XD-07) was deposited at Shanghai Institute of Materia Medica, Chinese Academy of Sciences, Shanghai, China.

1.3 Extraction and isolation

The frozen materials (943 g, dry weight) were cut into pieces and extracted exhaustively with Me_2CO at room temperature. The organic extract was evaporated to give a brown residue, which was partitioned between Et_2O and H_2O. The Et_2O solution was concentrated under reduced pressure to give a dark brown residue (36.3 g), which was fractionated by gradient Si gel (200−300 mesh) column chromatography (CC) (0 → 100% Et2O in petroleum ether (PE), yielding eight fractions (A−F). Fraction D was isolated by Sephadex LH-20 (PE/CH_2Cl_2/MeOH, 2:1:1), followed by silica gel CC (PE/CH_2Cl_2, 10:0 → 0:10) to give two subfractions (D2E and D2G). Fraction D2E was finally purified by reversed-phase HPLC (MeCN/H_2O, 70:30; 3.0 mL/min) to give compound **1** (11.6 mg, t_R = 17.1 min) and **4** (12.3 mg, t_R = 12.3 min), while compound **2** (1.2 mg, t_R = 18.6 min) was isolated from subfraction D2G by RP-HPLC (MeCN/H_2O, 60:40; 3.0 mL/min). Fraction E was fractioned by Sephadex LH-20 (PE/CH_2Cl_2/MeOH, 2:1:1), followed by silica gel CC (PE/Et_2O, 10:0 → 0:10) to give two subfractions (E3E and E3H). Subfraction E3E was further purified by stepwise HPLC (MeCN/H_2O, 62:38 → 70:30; 3.0 mL/min) to obtain compound **5** (17.0 mg, t_R = 5.3 min). Similarly, compound **3** (2.0 mg, t_R = 13.5 min) was isolated from fraction E3H by reversed-phase HPLC (MeCN/H2O, 75:25 → 98:2; 3.0 mL/min).

Ximaoornatin A (**1**): Colorless crystals; m.p. 166.2～166.4°C; $[\alpha]_D^{20}$ -46.7 (*c* 0.35, $CHCl_3$); IR (KBr) ν_{max}= 3445, 2919, 2849, 1959, 1620, 1384, 1156, 1043 cm^{-1}; ^1H and ^{13}C NMR data see Table 1; HR-ESIMS *m/z* 487.2670 $[M + Na]^+$ (calcd. for $C_{26}H_{40}NaO_7$, 487.2666).

Ximaoornatin B (**2**): Colorless oil; $[\alpha]_D^{20}$ -15.4 (*c* 0.12, $CHCl_3$); IR (KBr) ν_{max}= 3441, 2960, 2924, 2870, 1959, 1732, 1620, 1384, 1260, 1074, 1040 cm^{-1}; ^1H and ^{13}C NMR data see Table 1; HR-EIMS *m/z* 492.3080 $[M]^+$ (calcd. for $C_{28}H_{44}O_7$, 492.3082).

Ximaoornatin C (**3**): Colorless oil; $[\alpha]_D^{20}$ -55.0 (*c* 0.11, CH_3OH); IR (KBr) ν_{max}= 3444, 2924, 1959, 1731, 1620, 1384, 1260, 1045, 800 cm^{-1}; ^1H and ^{13}C NMR data see Table 1; HR-EIMS *m/z* 422.2665 $[M]^+$ (calcd. for $C_{24}H_{38}O_6$, 422.2663).

Litophynin K (**4**): Colorless oil; $[\alpha]_D^{20}$ -58.4 (*c* 0.24, $CHCl_3$); IR (KBr) ν_{max}= 3446, 2925, 1959, 1733, 1665, 1384, 1247, 1097, 1051 cm^{-1}; ^1H and ^{13}C NMR data see Table 1; HR-ESIMS *m/z* 471.2718 $[M + Na]^+$ (calcd. for $C_{26}H_{40}NaO_6$, 471.2717).

2. Original data for compounds 1–5

2.1 X-ray crystallographic analyses of 1

$C_{2.08}H_{3.2}O_{0.56}$, M_r = 37.17, monoclinic, crystal size 0.15 × 0.08 × 0.05 mm^3, space group $P2_1$, a = 11.0532(5) Å, b = 9.4225(4) Å, c = 12.3134(5) Å, V = 1271.54 (9) Å^3, Z = 25, D_{calcd} = 1.213 g/cm^3, $F(000)$ = 504.0, 19814 reflections measured (7.24° ≤ 2Θ ≤ 149.14°), 5096 unique (R_{int} = 0.0470, R_{sigma} = 0.0381) which were used in all calculations. The final R_1 was 0.0346 ($I > 2\sigma(I)$) and wR_2 was 0.0901 (all data). The X-ray measurements were made on a Bruker D8 Venture X-ray diffractometer with with Cu Kα radiation (λ=1.54178 Å) at 170.0 K. The structure was solved with the ShelXT[1] structure solution program using Intrinsic Phasing and refined with the ShelXL[2] refinement package using Least Squares minimisation. Crystallographic data for **1** has been deposited at the Cambridge Crystallographic Data Centre (Deposition nos. CCDC 2126980). Copies of these data can be obtained free of charge via www.ccdc.cam.ac.uk/conts/retrieving.html or from the Cambridge Crystallographic Data Centre, 12 Union Road, Cambridge CB21EZ, UK. [Fax: (+44) 1223-336-033. E-mail: deposit@ccdc.cam.ac.uk].

References

(1) Sheldrick, G.M. *Acta Cryst.* **2015**, *A71*, 3-8.
(2) Sheldrick, G.M. *Acta Cryst.* **2015**, *C71*, 3-8.

Table S1. X-ray crystallographic data for **1**.

Empirical formula	$C_{2.08}H_{3.2}O_{0.56}$
Formula weight	37.17
Temperature/K	170.0
Crystal system	monoclinic
Space group	$P2_1$
a/Å	11.0532(5)
b/Å	9.4225(4)
c/Å	12.3134(5)
α/°	90
β/°	97.470(2)
γ/°	90
Volume/Å^3	1271.54 (9)
Z	25
ρ_{calc}g/cm^3	1.213
μ/mm^{-1}	0.706
F(000)	504.0
Crystal size/mm^3	0.15 × 0.08 × 0.05
Radiation	Cu *K*α (λ = 1.54178)
2Θ range for data collection/°	7.24 to 149.14
Index ranges	$-13 \leq h \leq 13$, $-11 \leq k \leq 11$, $-15 \leq l \leq 15$
Reflections collected	19814
Independent reflections	5096 [Rint = 0.0470, Rsigma = 0.0381]
Data/restraints/parameters	5096/1/305
Goodness-of-fit on F^2	1.054
Final R indexes [I>=2σ (I)]	R1 = 0.0346, wR_2 =0.0878
Final R indexes [all data]	R1 = 0.0376, wR_2 = 0.0901
Largest diff. peak/hole / e Å^{-3}	0.25/-0.15
Flack parameter	-0.09(7)

2.2 NMR, MS, and IR spectra of compounds 1−5

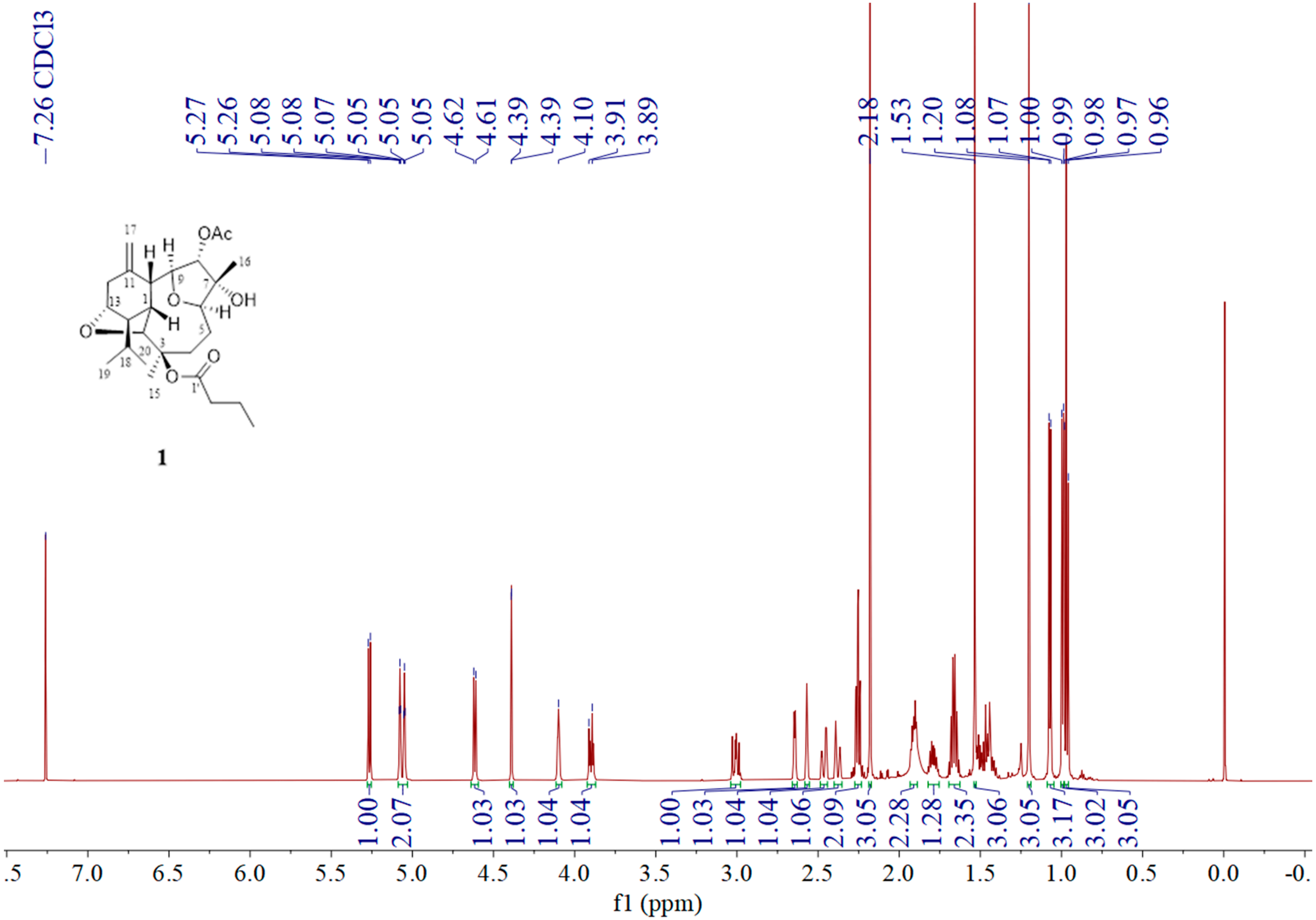

Figure S1. ^{1}H NMR spectrum of ximaoornatin A (**1**) in CDCl3.

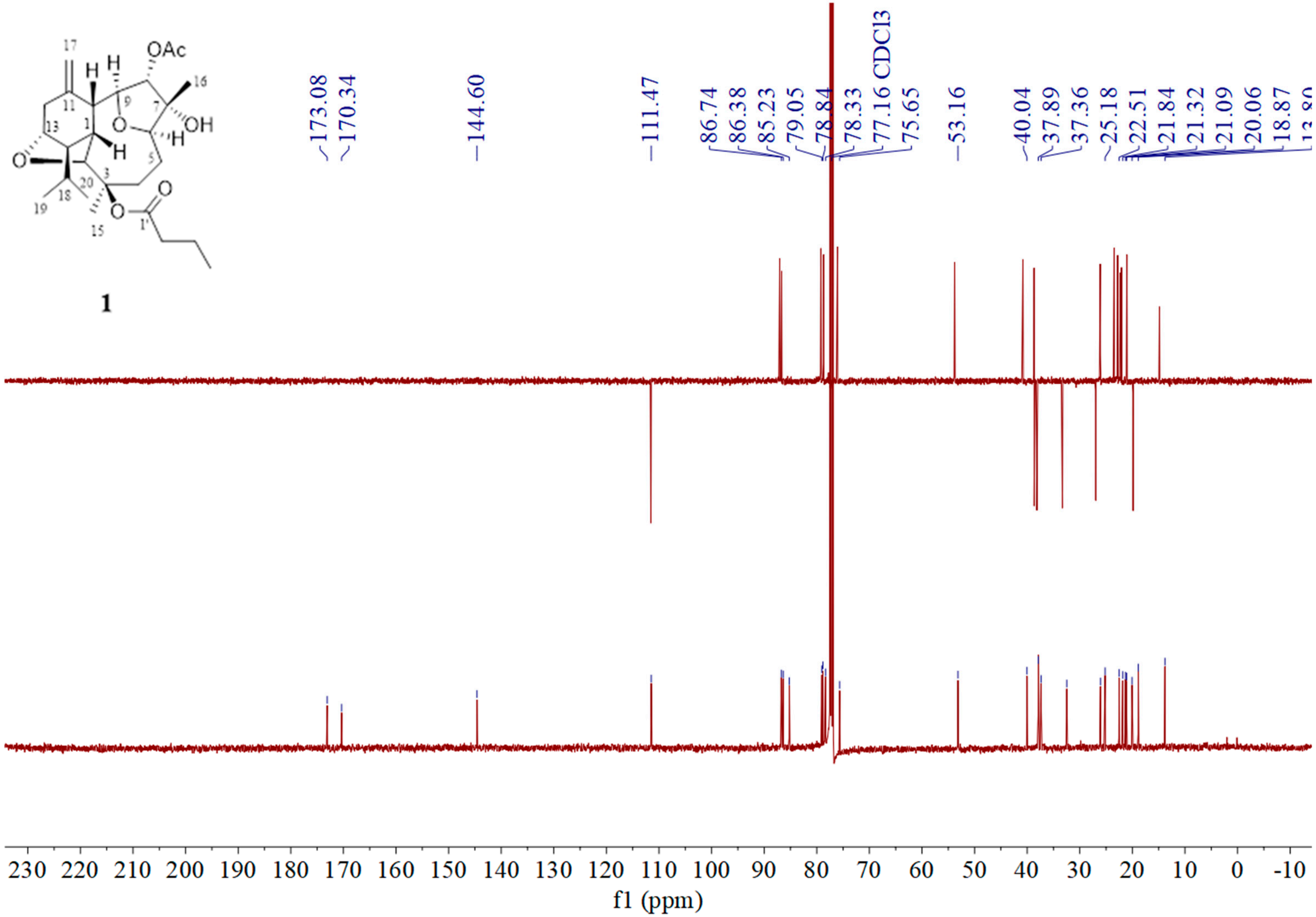

Figure S2. ^{13}C NMR spectrum of ximaoornatin A (**1**) in $CDCl_3$.

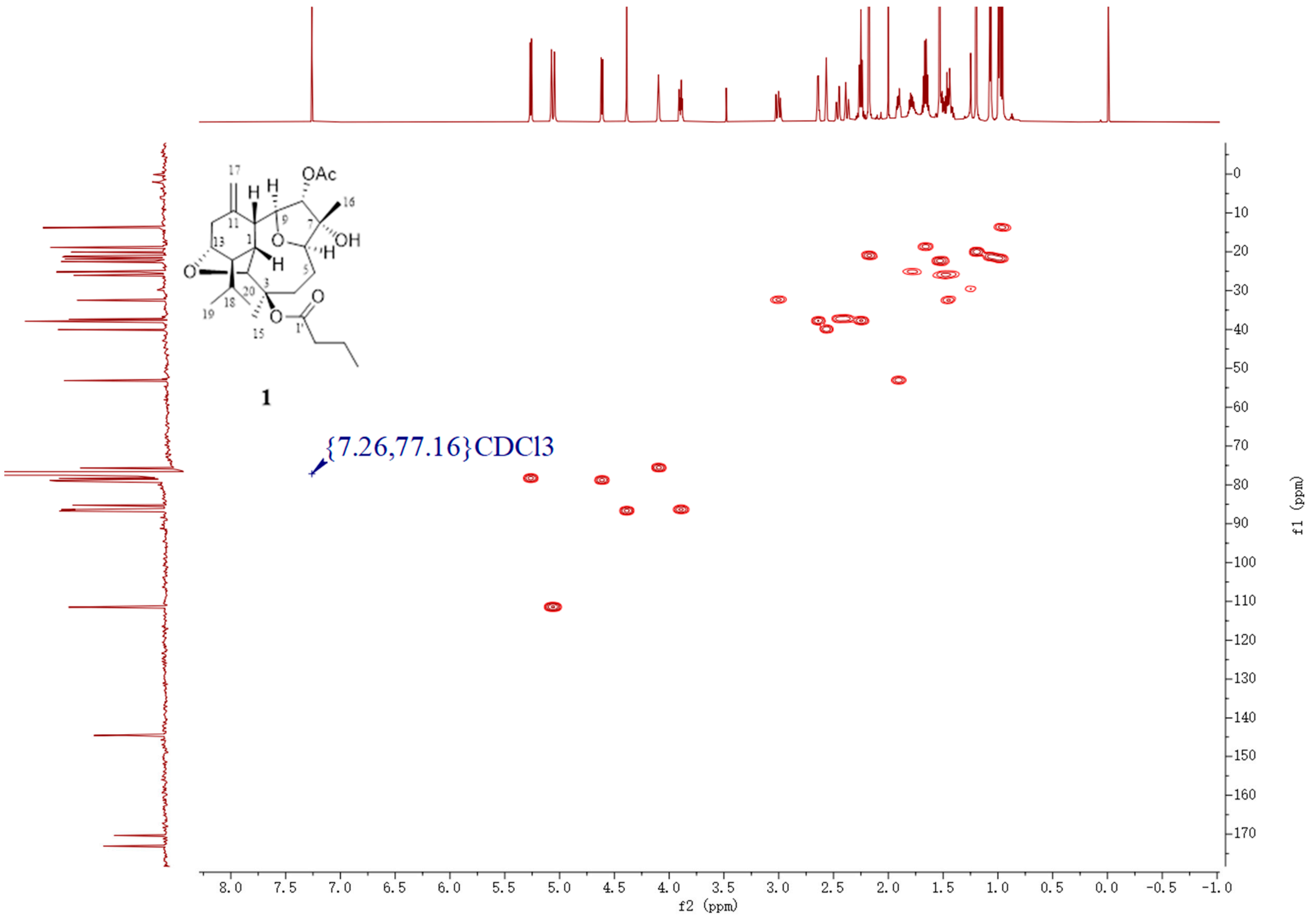

Figure S3. HSQC spectrum of ximaoornatin A (**1**) in $CDCl_3$.

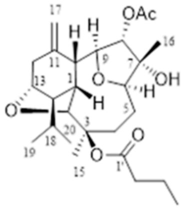

1

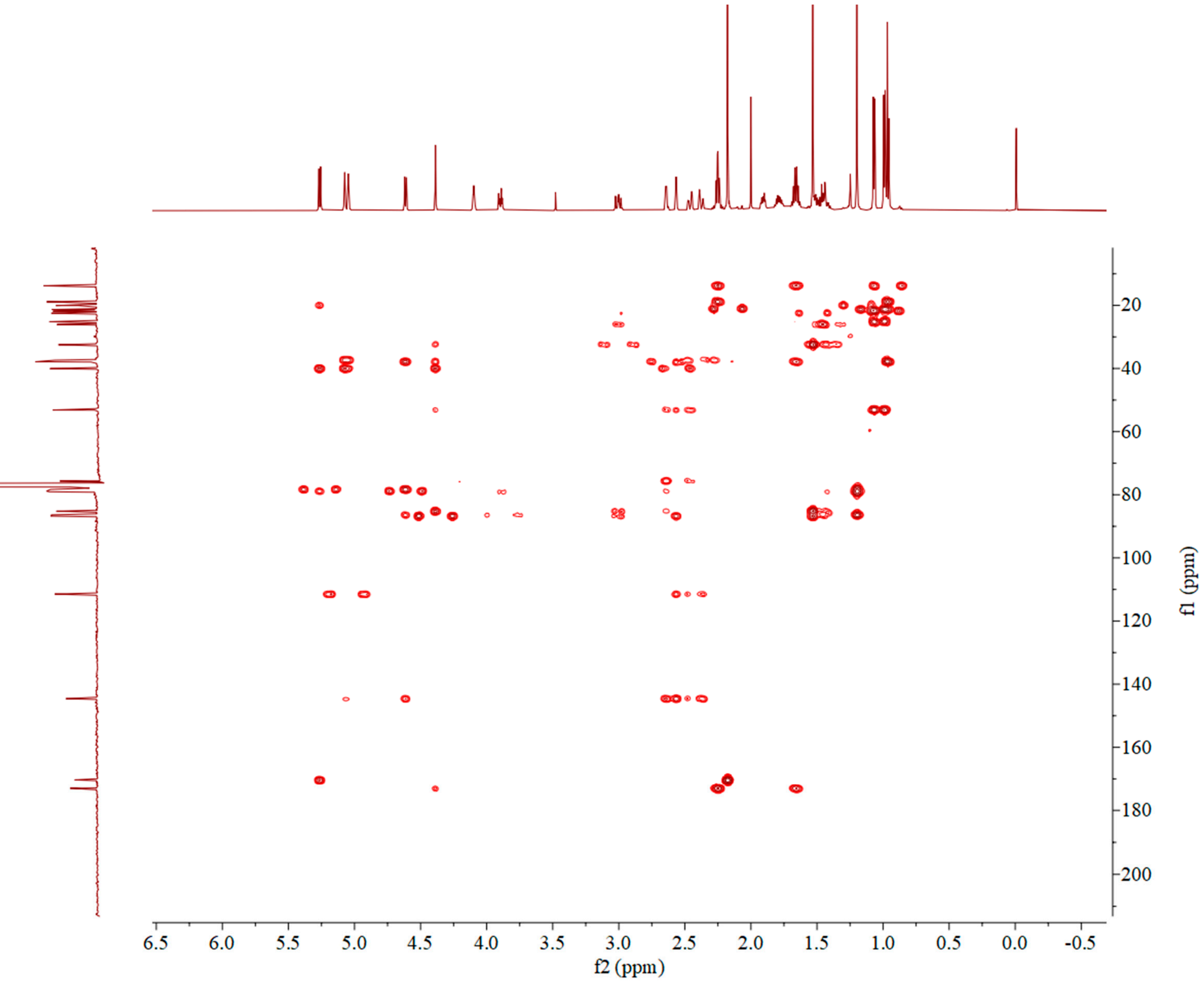

Figure S4. HMBC spectrum of ximaoornatin A (**1**) in $CDCl_3$.

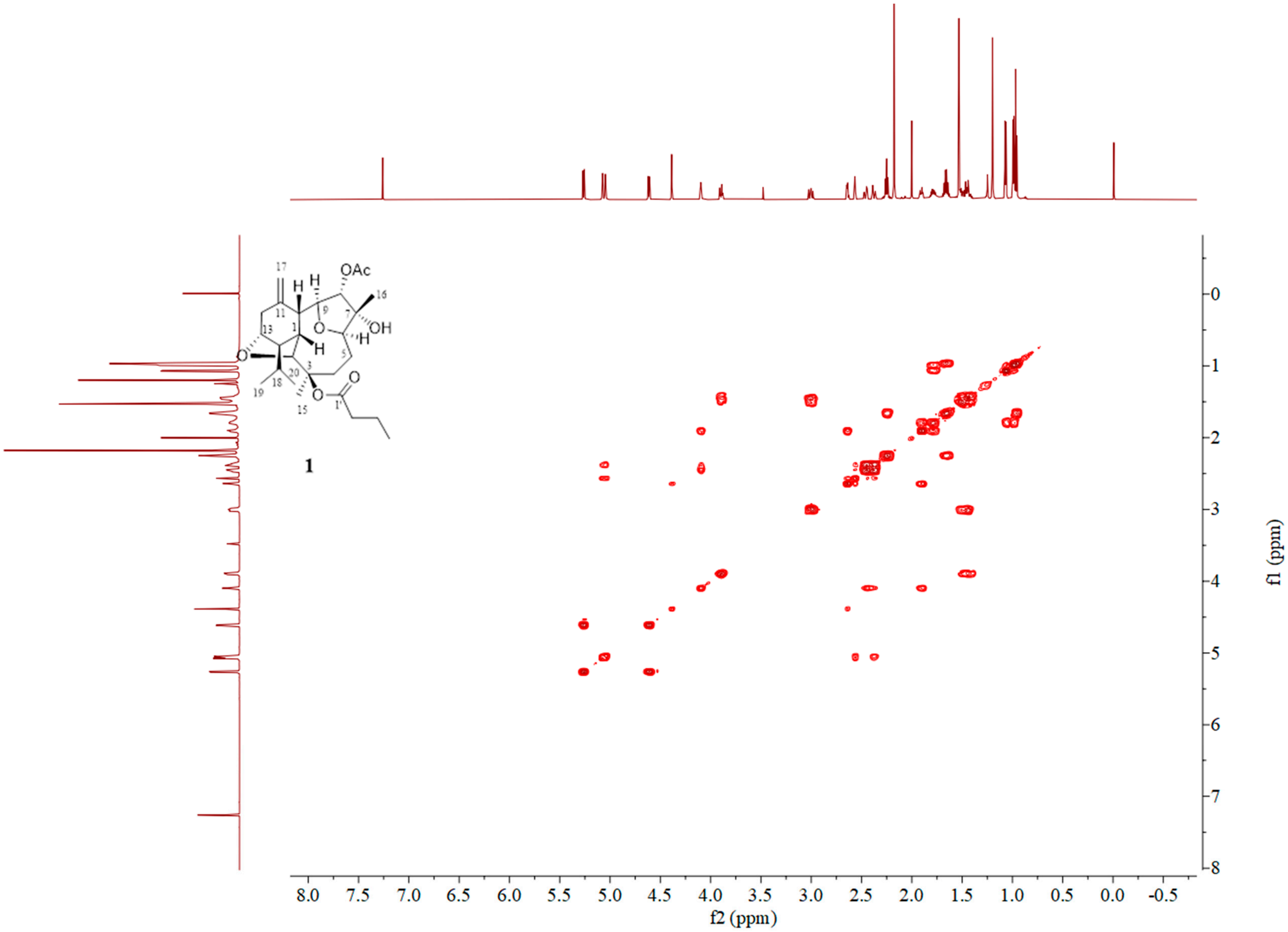

Figure S5. ¹H-¹H COSY spectrum of ximaoornatin A (**1**) in $CDCl_3$.

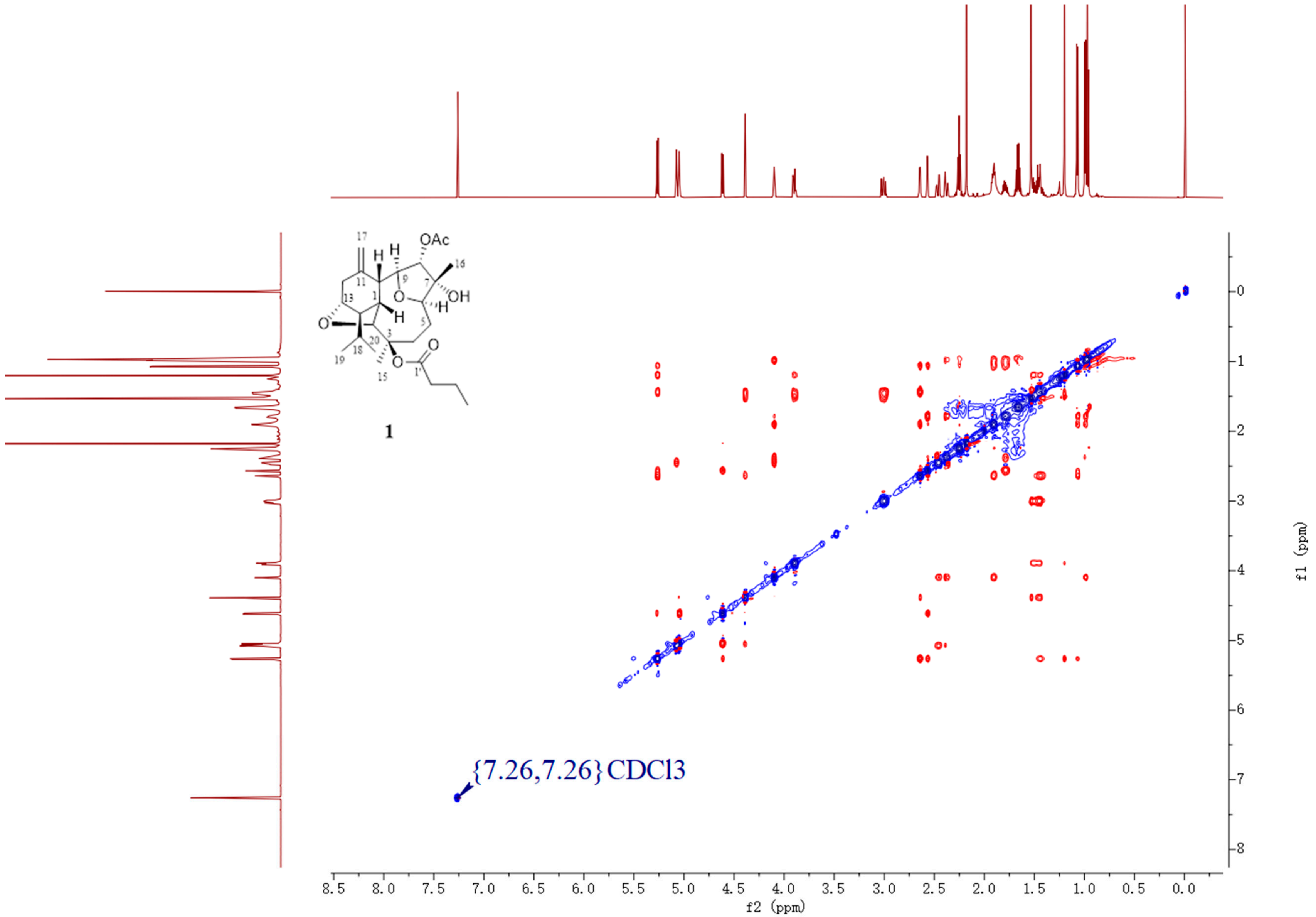

Figure S6. NOESY spectrum of ximaoornatin A (**1**) in $CDCl_3$.

Qualitative Analysis Report

**Data Filename**	ESIH_20200526_GYW_SLL_04.d	**Sample Name**	A8-DBE-4
**Sample Type**	Sample	**Position**	P1-D4
**Instrument Name**	Agilent G6520 Q-TOF	**Acq Method**	20160322_MS_ESIH_POS_1min.m
**Acquired Time**	5/26/2020 17:44:19	**IRM Calibration Status**	Success
**DA Method**	small molecular data analysis method.m	**Comment**	ESIH by ZZY

User Spectra

Fragmentor Voltage	Collision Energy	Ionization Mode
135	0	ESI

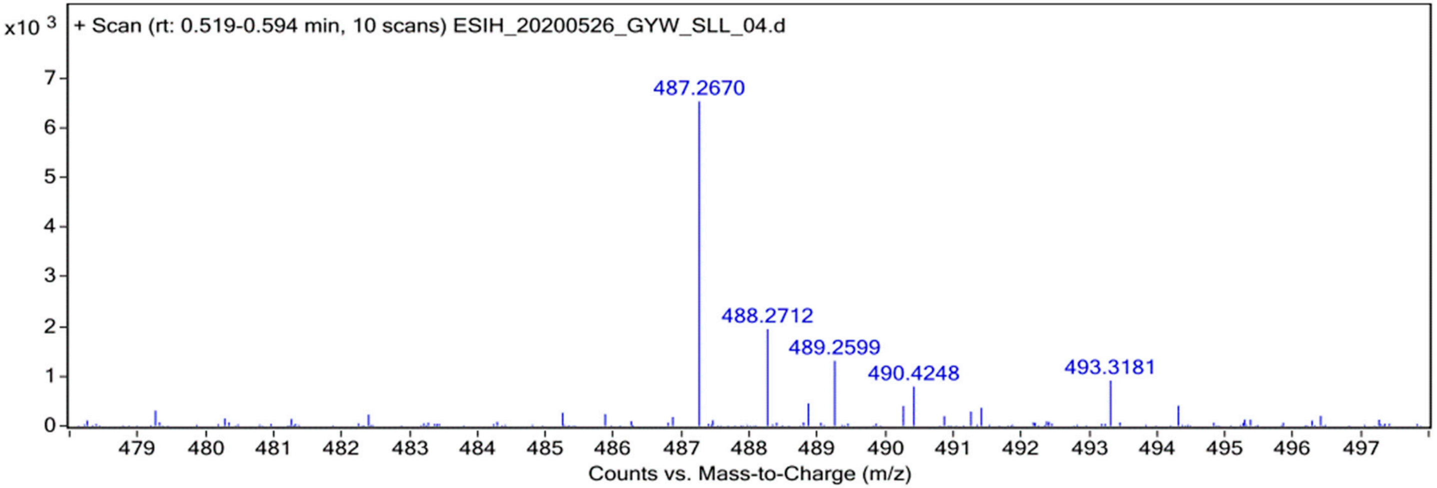

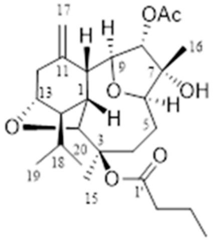

1

Formula Calculator Results

m/z	Calc m/z	Diff (mDa)	Diff (ppm)	Ion Formula	Ion
487.267	487.2666	-0.39	-0.79	C26 H40 Na O7	(M+Na)+

--- End Of Report ---

Figure S7. HRESIMS spectrum of ximaoornatin A (**1**) in MeOH.

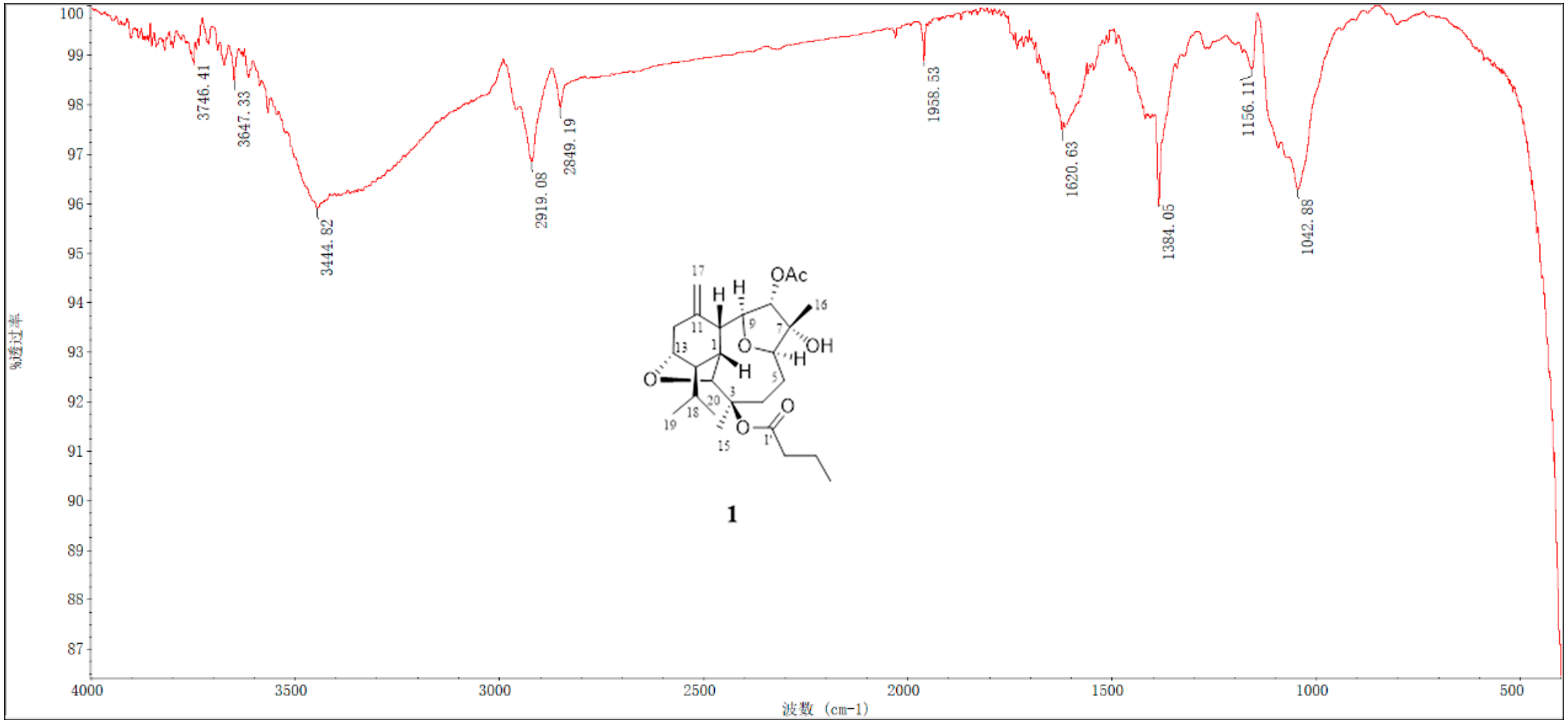

1

Figure S8. IR spectrum of ximaoornatin A (**1**).

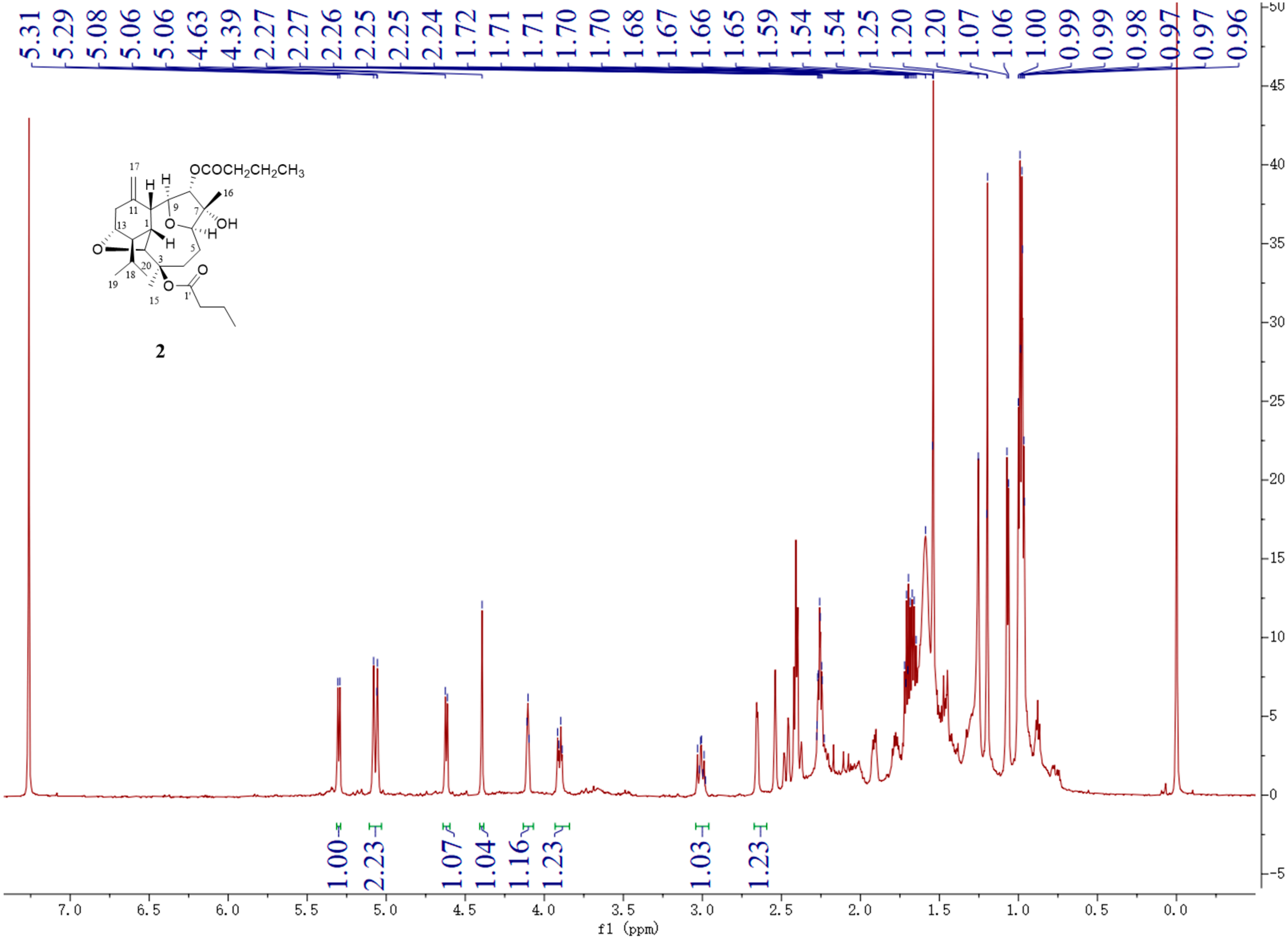

Figure S9. ^{1}H NMR spectrum of ximaoornatin B (**2**) in $CDCl_3$.

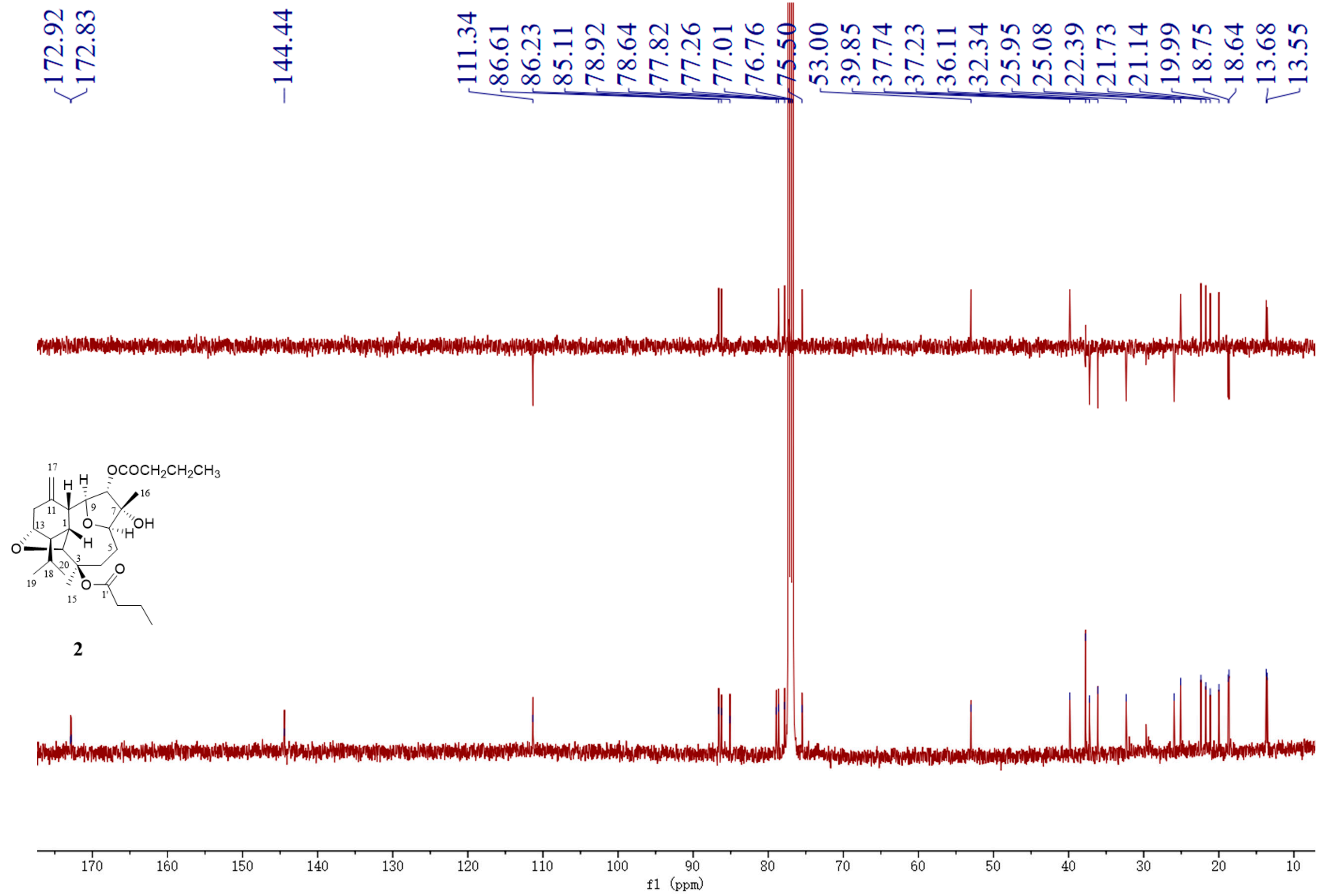

Figure S10. ^{13}C NMR spectrum of ximaoornatin B (**2**) in $CDCl_3$.

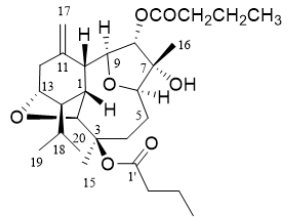

2

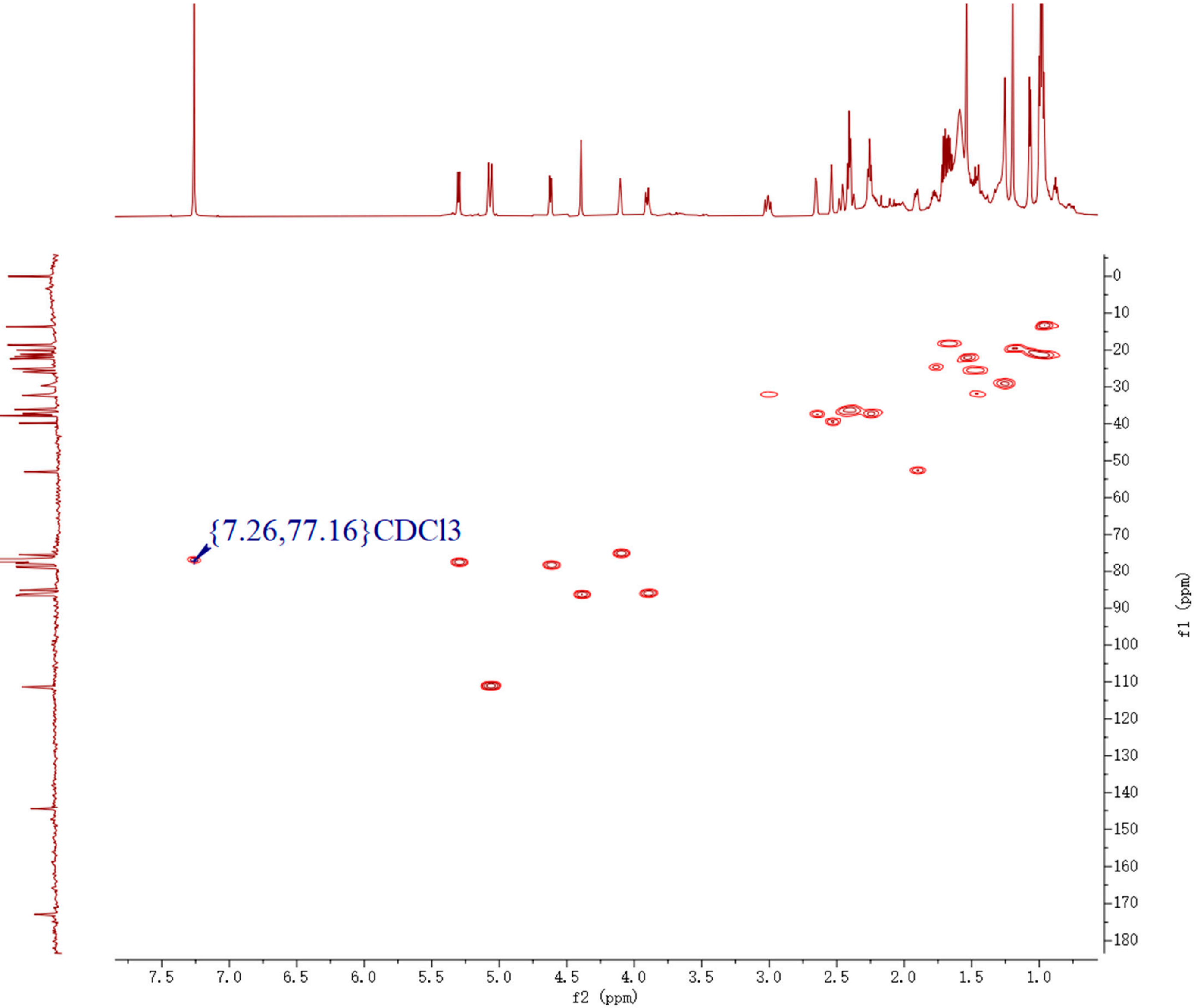

Figure S11. HSQC spectrum of ximaoornatin B (**2**) in $CDCl_3$.

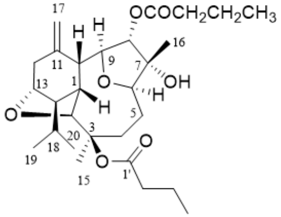

2

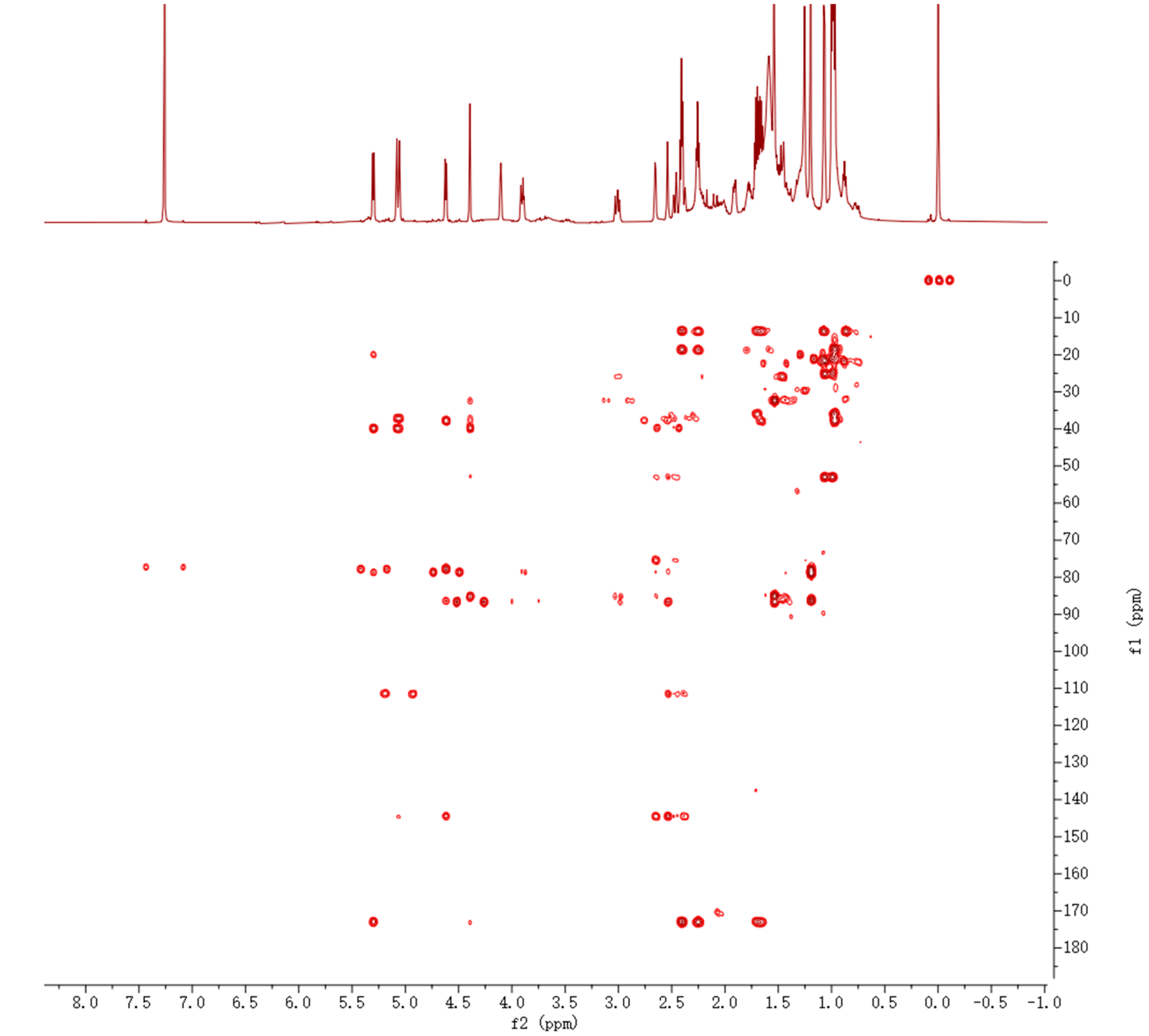

Figure S12. HMBC spectrum of ximaoornatin B (**2**) in $CDCl_3$.

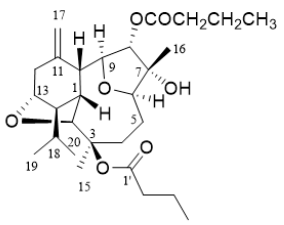

2

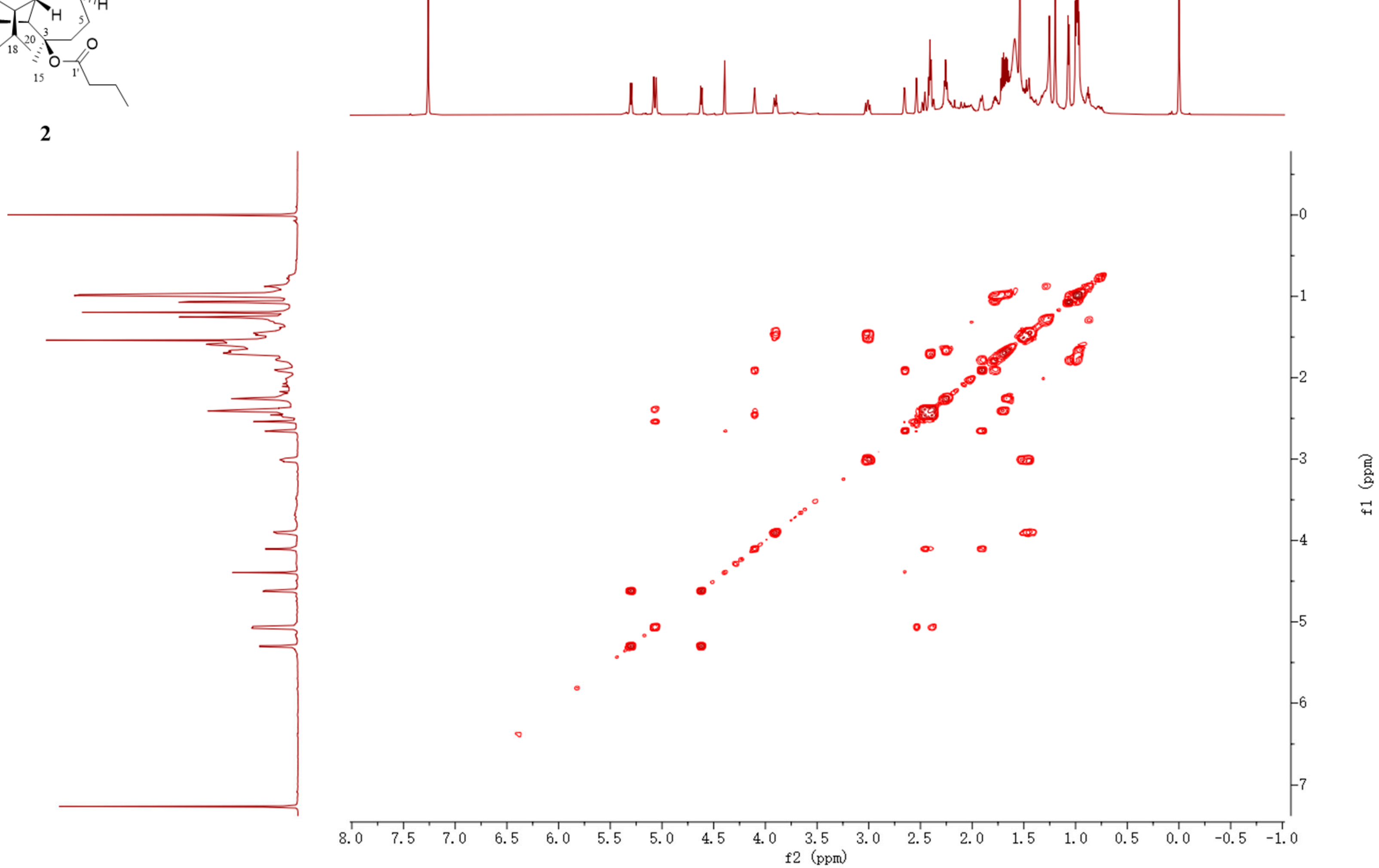

Figure S13. ^{1}H-^{1}H COSY spectrum of ximaoornatin B (**2**) in $CDCl_3$.

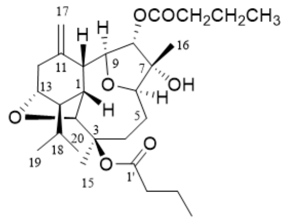

2

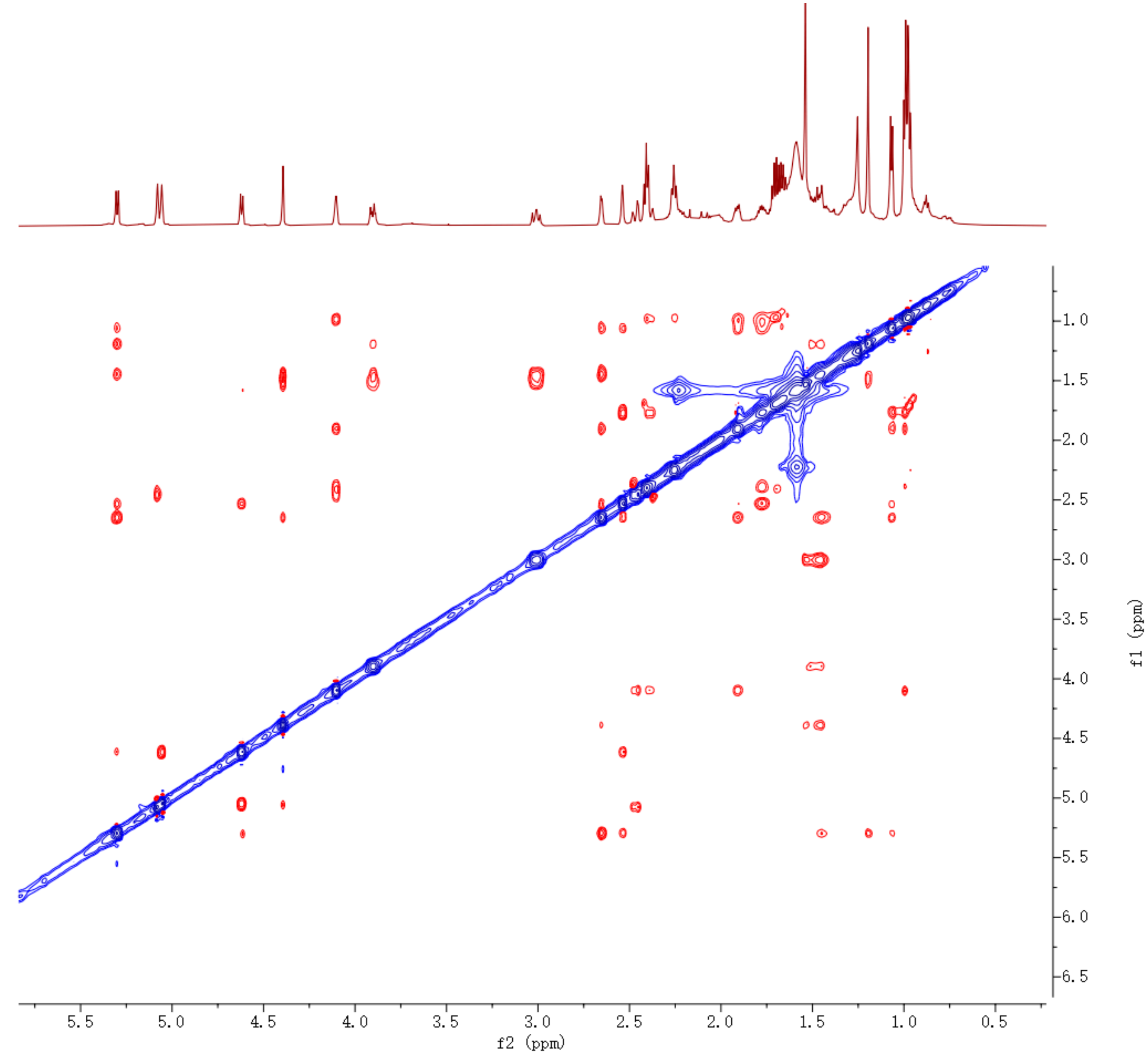

Figure S14. NOESY spectrum of ximaoornatin B (**2**) in $CDCl_3$.

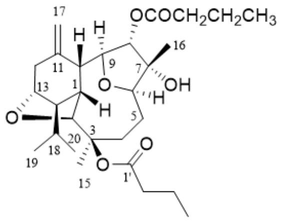

2

EIH-20200605-GYW-SLL-04_A8-DBG-6 -c1 6/9/2020 3:02:21 PM

EIH-20200605-GYW-SLL-04_A8-DBG-6 -c1#4 RT: 4.14
T: + c EI Full ms [49.50-800.50]
m/z= 48-803

m/z	Intensity	Relative	Theo. Mass	Delta (mmu)	RDB equiv.	Compositior
258.1601	81614.0	1.13	258.1614	-1.38	7.0	$C_{17}H_{22}O_2$
259.1690	119677.0	1.65	259.1693	-0.27	6.5	$C_{17}H_{23}O_2$
260.1778	67539.0	0.93	260.1771	0.72	6.0	$C_{17}H_{24}O_2$
261.1869	89138.0	1.23	261.1849	1.95	5.5	$C_{17}H_{25}O_2$
262.1907	41028.0	0.57	262.1927	-1.98	5.0	$C_{17}H_{26}O_2$
263.1650	33283.0	0.46	263.1642	0.83	5.5	$C_{16}H_{23}O_3$
269.1898	80950.0	1.12	269.1900	-0.16	7.5	$C_{19}H_{25}O_1$
270.1986	40276.0	0.56	270.1978	0.74	7.0	$C_{19}H_{26}O_1$
271.2057	52889.0	0.73	271.2056	0.07	6.5	$C_{19}H_{27}O_1$
273.1498	96175.0	1.33	273.1485	1.30	7.5	$C_{17}H_{21}O_3$
273.1851	146454.0	2.02	273.1849	0.19	6.5	$C_{18}H_{25}O_2$
275.1640	56829.0	0.79	275.1642	-0.16	6.5	$C_{17}H_{23}O_3$
277.1805	66610.0	0.92	277.1798	0.67	5.5	$C_{17}H_{25}O_3$
282.0516	56342.0	0.78	282.0523	-0.68	12.0	$C_{16}H_{10}O_5$
283.1698	45188.0	0.62	283.1693	0.57	8.5	$C_{19}H_{23}O_2$
287.1994	143267.0	1.98	287.2006	-1.13	6.5	$C_{19}H_{27}O_2$
288.2063	76613.0	1.06	288.2084	-2.05	6.0	$C_{19}H_{28}O_2$
289.2164	39700.0	0.55	289.2162	0.15	5.5	$C_{19}H_{29}O_2$
297.1848	87589.0	1.21	297.1849	-0.15	8.5	$C_{20}H_{25}O_2$
298.1914	72098.0	1.00	298.1927	-1.30	8.0	$C_{20}H_{26}O_2$
299.2004	94006.0	1.30	299.2006	-0.14	7.5	$C_{20}H_{27}O_2$
300.2076	106266.0	1.47	300.2084	-0.82	7.0	$C_{20}H_{28}O_2$
301.1818	56829.0	0.79	301.1798	1.98	7.5	$C_{19}H_{25}O_3$
301.2154	54394.0	0.75	301.2162	-0.82	6.5	$C_{20}H_{29}O_2$
302.2222	33814.0	0.47	302.2240	-1.79	6.0	$C_{20}H_{30}O_2$
305.2132	44967.0	0.62	305.2111	2.10	5.5	$C_{19}H_{29}O_3$
315.1955	497918.0	6.88	315.1955	0.04	7.5	$C_{20}H_{27}O_3$
316.2015	517481.0	7.15	316.2033	-1.79	7.0	$C_{20}H_{28}O_3$
317.2096	209966.0	2.90	317.2111	-1.53	6.5	$C_{20}H_{29}O_3$
318.2185	84402.0	1.17	318.2189	-0.42	6.0	$C_{20}H_{30}O_3$
319.2253	45587.0	0.63	319.2268	-1.50	5.5	$C_{20}H_{31}O_3$
333.2065	244356.0	3.38	333.2060	0.51	6.5	$C_{20}H_{29}O_4$
334.2114	102637.0	1.42	334.2139	-2.51	6.0	$C_{20}H_{30}O_4$
342.0154	37399.0	0.52	342.0159	-0.48	18.0	$C_{20}H_6O_6$
369.1252	36425.0	0.50	369.1274	-2.17	20.5	$C_{28}H_{17}O_1$
404.2558	2630425.0	36.35	404.2557	0.07	7.0	$C_{24}H_{36}O_5$
421.2605	219925.0	3.04	421.2585	2.03	6.5	$C_{24}H_{37}O_6$
422.2669	92989.0	1.29	422.2663	0.62	6.0	$C_{24}H_{38}O_6$
431.0887	35097.0	0.49	431.0914	-2.71	21.5	$C_{28}H_{15}O_5$
492.3080	39700.0	0.55	492.3082	-0.14	7.0	$C_{28}H_{44}O_7$

Figure S15. HREIMS spectrum of ximaoornatin B (**2**) in MeOH.

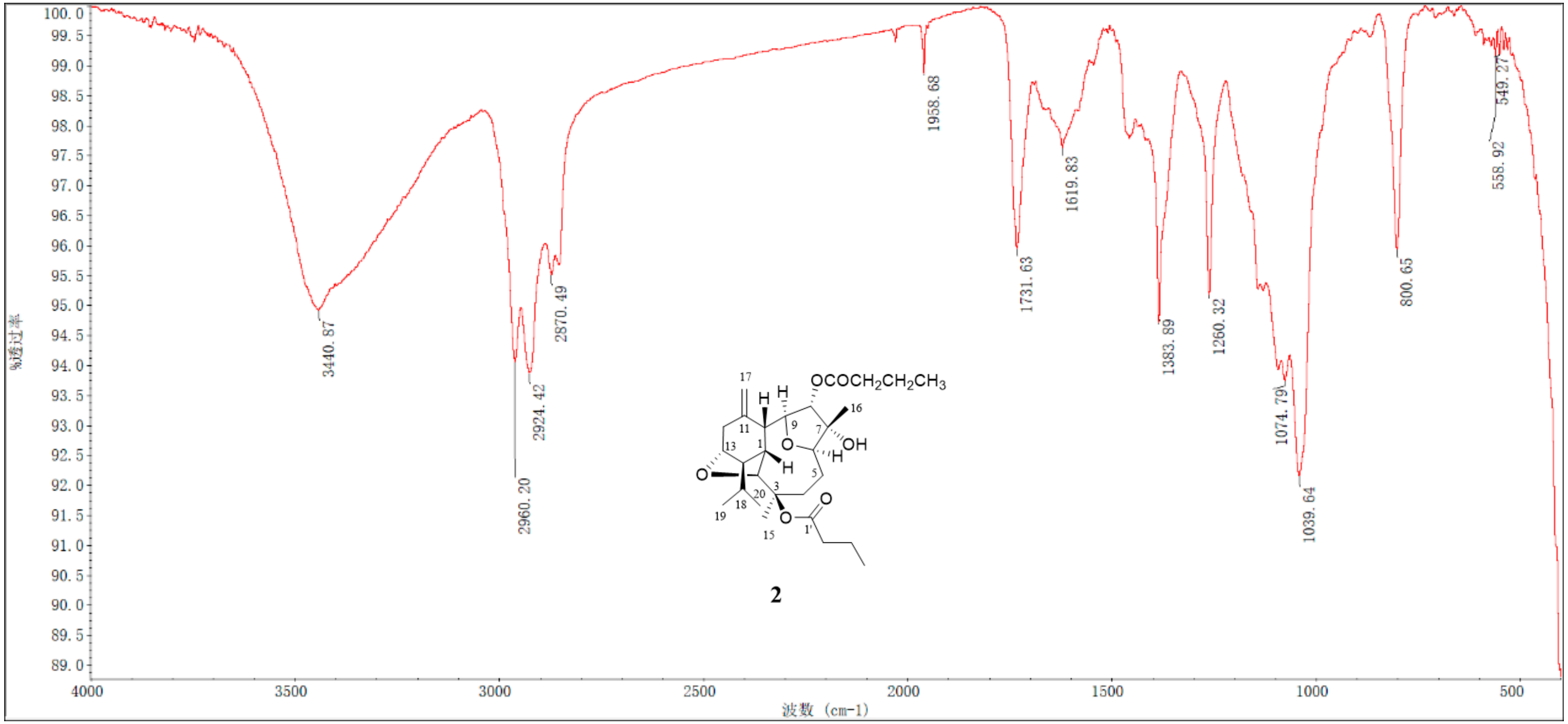

Figure S16. IR spectrum of ximaoornatin B (**2**).

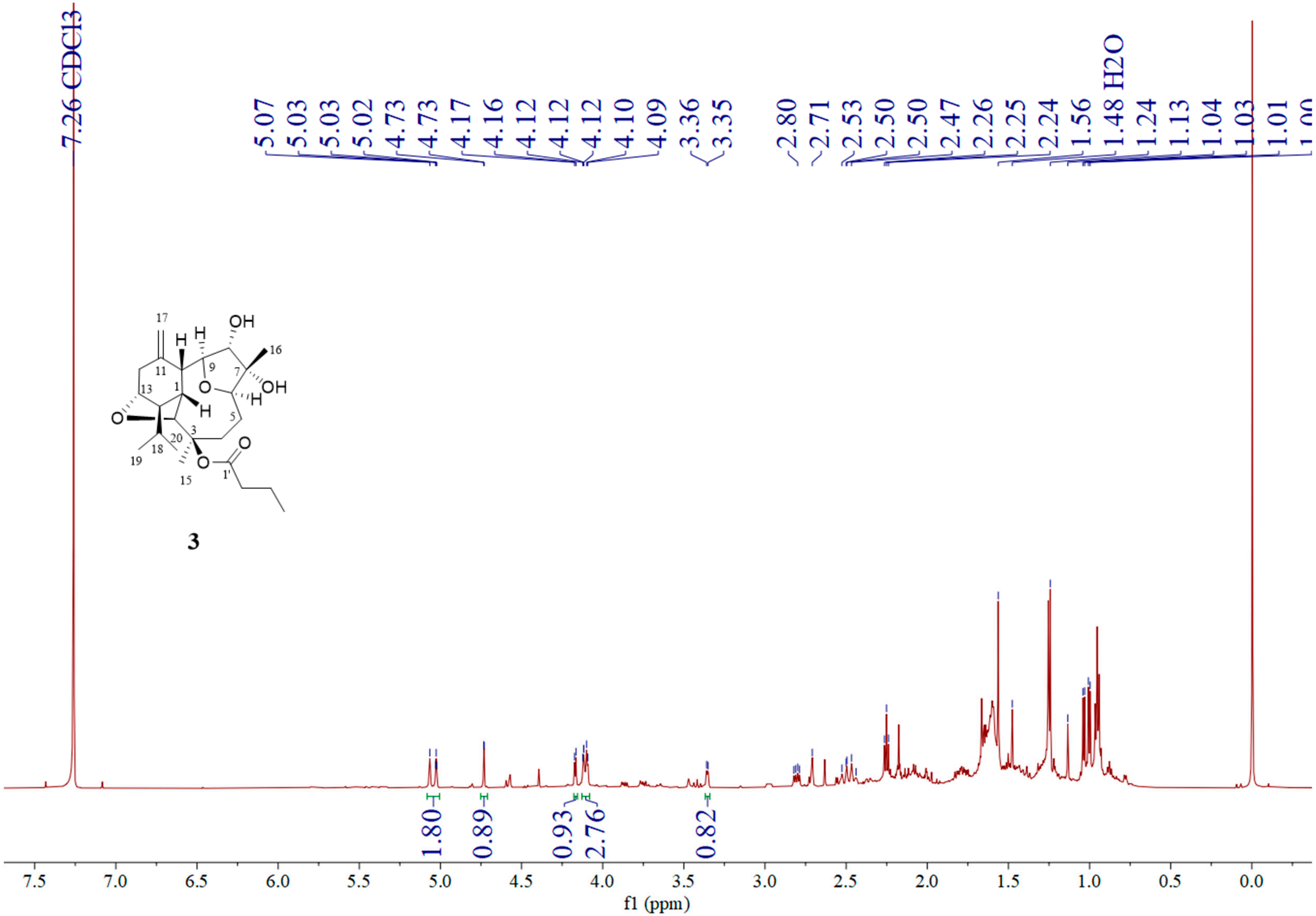

Figure S17. ^{1}H NMR spectrum of ximaoornatin C (**3**) in $CDCl_3$.

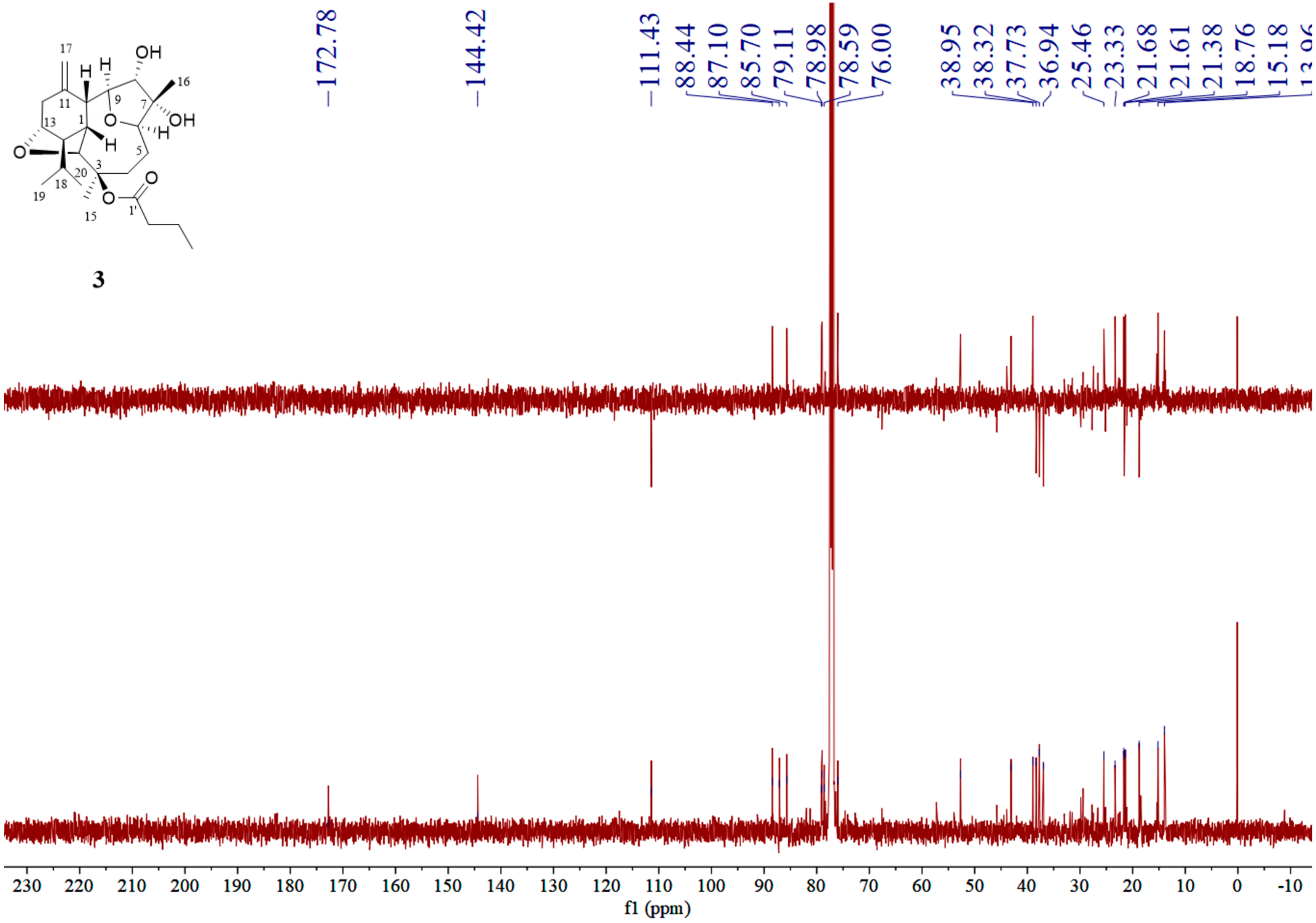

Figure S18. ^{13}C NMR spectrum of ximaoornatin C (**3**) in $CDCl_3$.

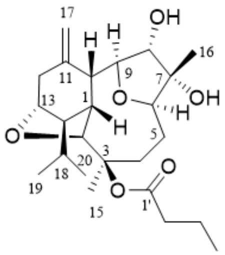

3

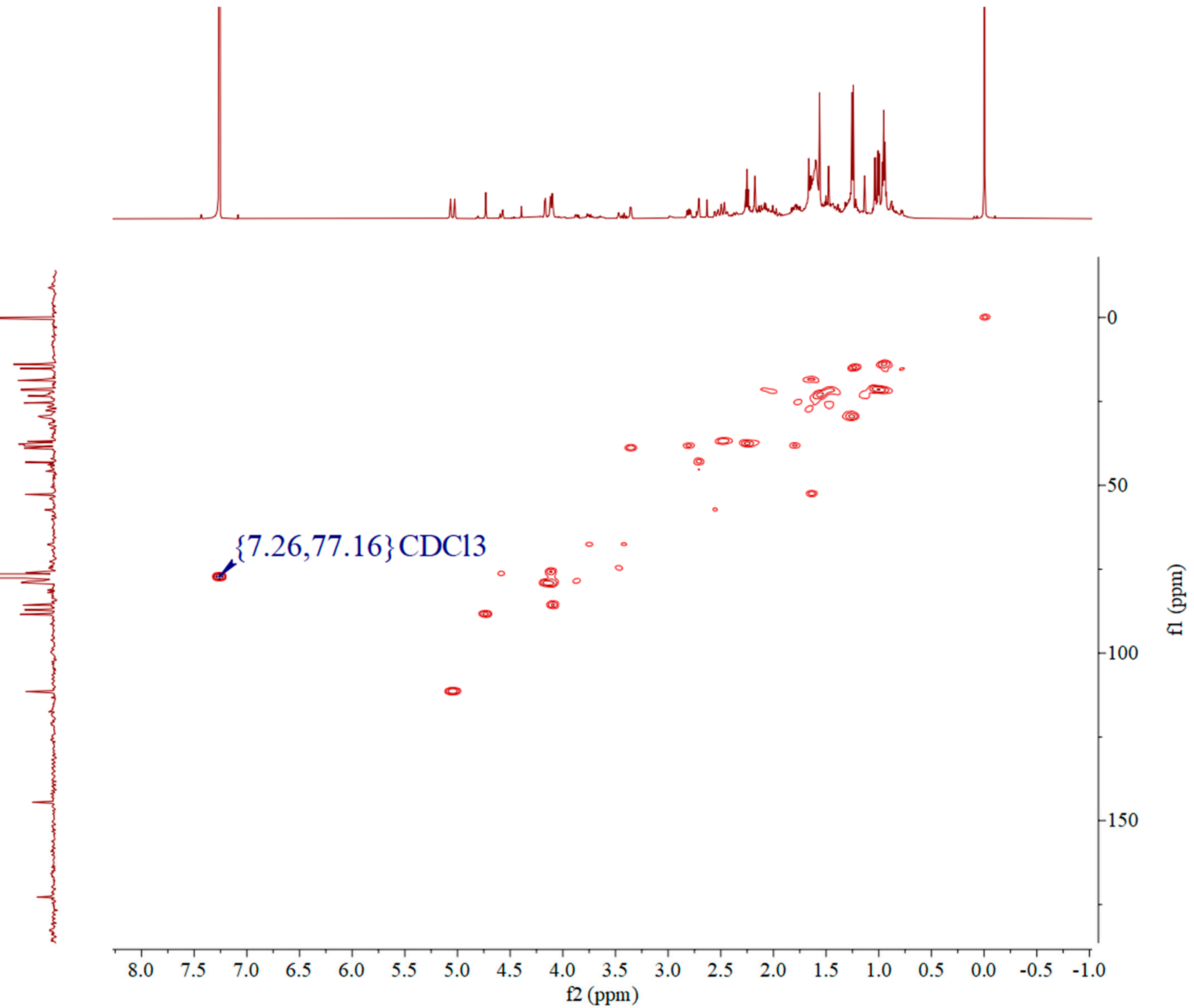

Figure S19. HSQC spectrum of ximaoornatin C (**3**) in $CDCl_3$.

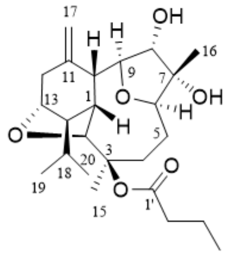

3

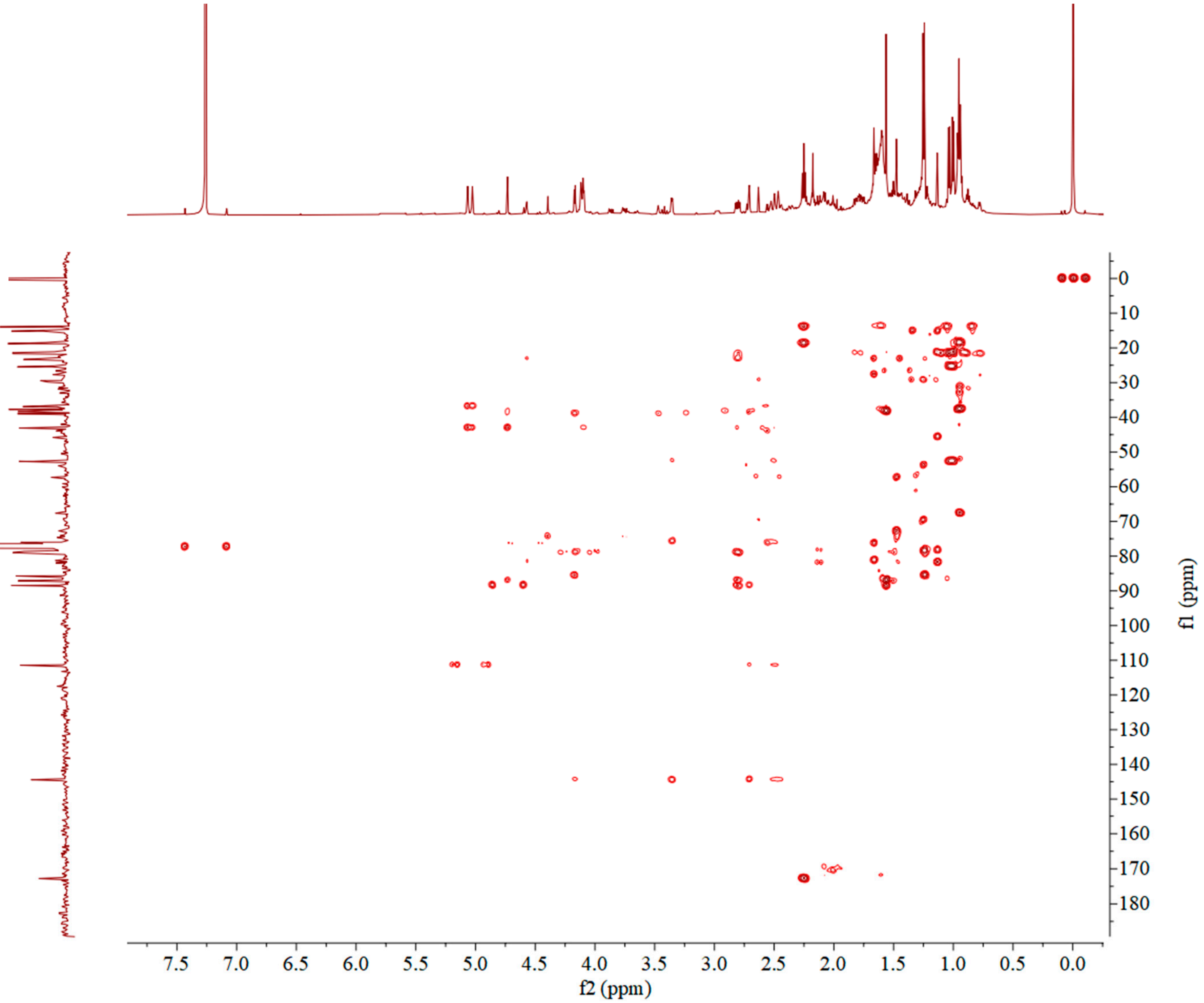

Figure S20. HMBC spectrum of ximaoornatin C (**3**) in $CDCl_3$.

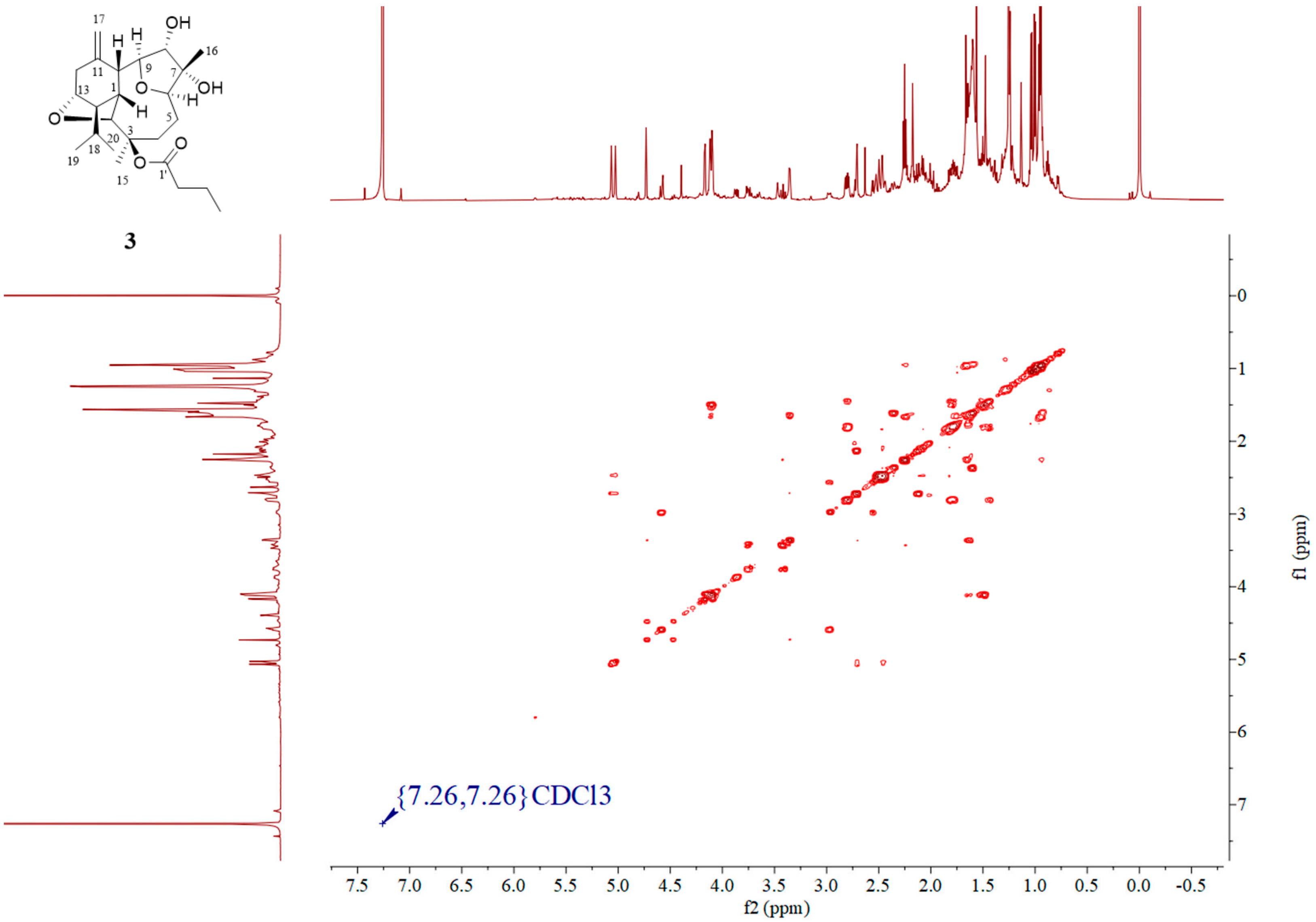

Figure S21. ^{1}H-^{1}H COSY spectrum of ximaoornatin C (**3**) in $CDCl_3$.

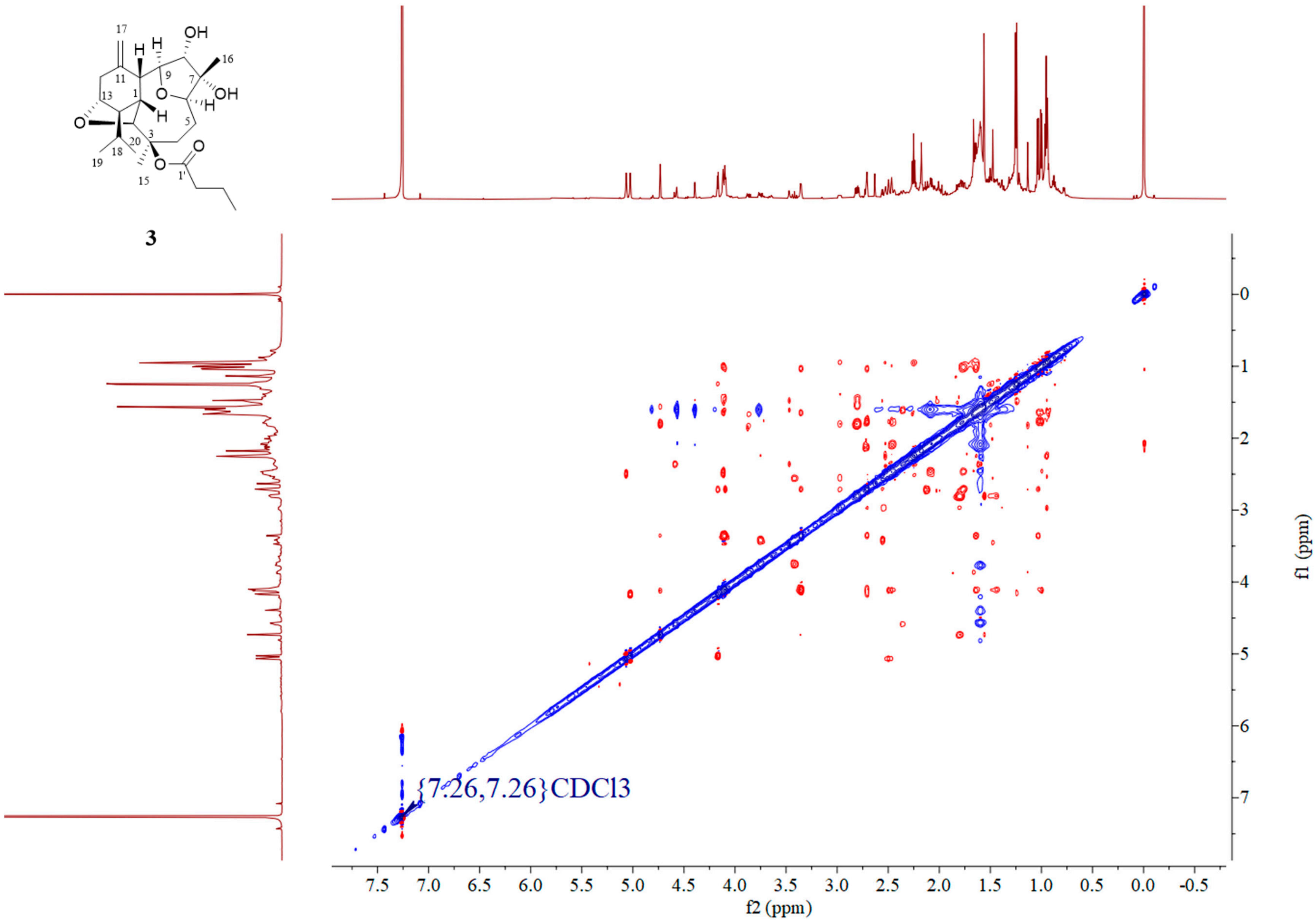

Figure S22. NOESY spectrum of ximaoornatin C (**3**) in $CDCl_3$.

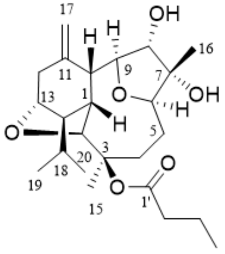

3

D:\data\2021\EI202101422_A8-ECH-3 -c1 7/15/2021 1:04:39 PM

EI202101422_A8-ECH-3 -c1#13 RT: 2.48
T: + c EI Full ms [49.50-800.50]
m/z= 48-803

m/z	Intensity	Relative	Theo. Mass	Delta (mmu)	RDB equiv.	Composition
258.1588	161917.0	0.61	258.1614	-2.65	7.0	$C_{17}H_{22}O_2$
259.1683	328343.0	1.24	259.1693	-0.92	6.5	$C_{17}H_{23}O_2$
260.1770	202174.0	0.77	260.1771	-0.05	6.0	$C_{17}H_{24}O_2$
261.1492	165183.0	0.63	261.1485	0.67	6.5	$C_{16}H_{21}O_3$
261.1851	176011.0	0.67	261.1849	0.21	5.5	$C_{17}H_{25}O_2$
263.1650	320355.0	1.21	263.1642	0.88	5.5	$C_{16}H_{23}O_3$
273.1481	335621.0	1.27	273.1485	-0.47	7.5	$C_{17}H_{21}O_3$
273.1847	325503.0	1.23	273.1849	-0.21	6.5	$C_{18}H_{25}O_2$
275.1641	268063.0	1.01	275.1642	-0.04	6.5	$C_{17}H_{23}O_3$
277.1802	158580.0	0.60	277.1798	0.35	5.5	$C_{17}H_{25}O_3$
279.1592	561510.0	2.12	279.1591	0.10	5.5	$C_{16}H_{23}O_4$
287.2012	272288.0	1.03	287.2006	0.65	6.5	$C_{19}H_{27}O_2$
289.1800	160639.0	0.61	289.1798	0.16	6.5	$C_{18}H_{25}O_3$
291.1601	172567.0	0.65	291.1591	1.05	6.5	$C_{17}H_{23}O_4$
291.1952	277400.0	1.05	291.1955	-0.27	5.5	$C_{18}H_{27}O_3$
293.1746	168343.0	0.64	293.1747	-0.16	5.5	$C_{17}H_{25}O_4$
299.2001	170402.0	0.64	299.2006	-0.49	7.5	$C_{20}H_{27}O_2$
305.2109	512661.0	1.94	305.2111	-0.26	5.5	$C_{19}H_{29}O_3$
306.2162	179916.0	0.68	306.2189	-2.73	5.0	$C_{19}H_{30}O_3$
315.1954	509928.0	1.93	315.1955	-0.05	7.5	$C_{20}H_{27}O_3$
316.2024	352661.0	1.33	316.2033	-0.91	7.0	$C_{20}H_{28}O_3$
317.2099	209701.0	0.79	317.2111	-1.22	6.5	$C_{20}H_{29}O_3$
333.2054	368565.0	1.39	333.2060	-0.68	6.5	$C_{20}H_{29}O_4$
334.2131	428632.0	1.62	334.2139	-0.75	6.0	$C_{20}H_{30}O_4$
335.2202	575994.0	2.18	335.2217	-1.46	5.5	$C_{20}H_{31}O_4$
351.2168	1492544.0	5.65	351.2166	0.15	5.5	$C_{20}H_{31}O_5$
356.0702	217049.0	0.82	356.0679	2.28	17.0	$C_{22}H_{12}O_5$
422.2665	179206.0	0.68	422.2663	0.24	6.0	$C_{24}H_{38}O_6$

Figure S23. HREIMS spectrum of ximaoornatin C (**3**) in MeOH.

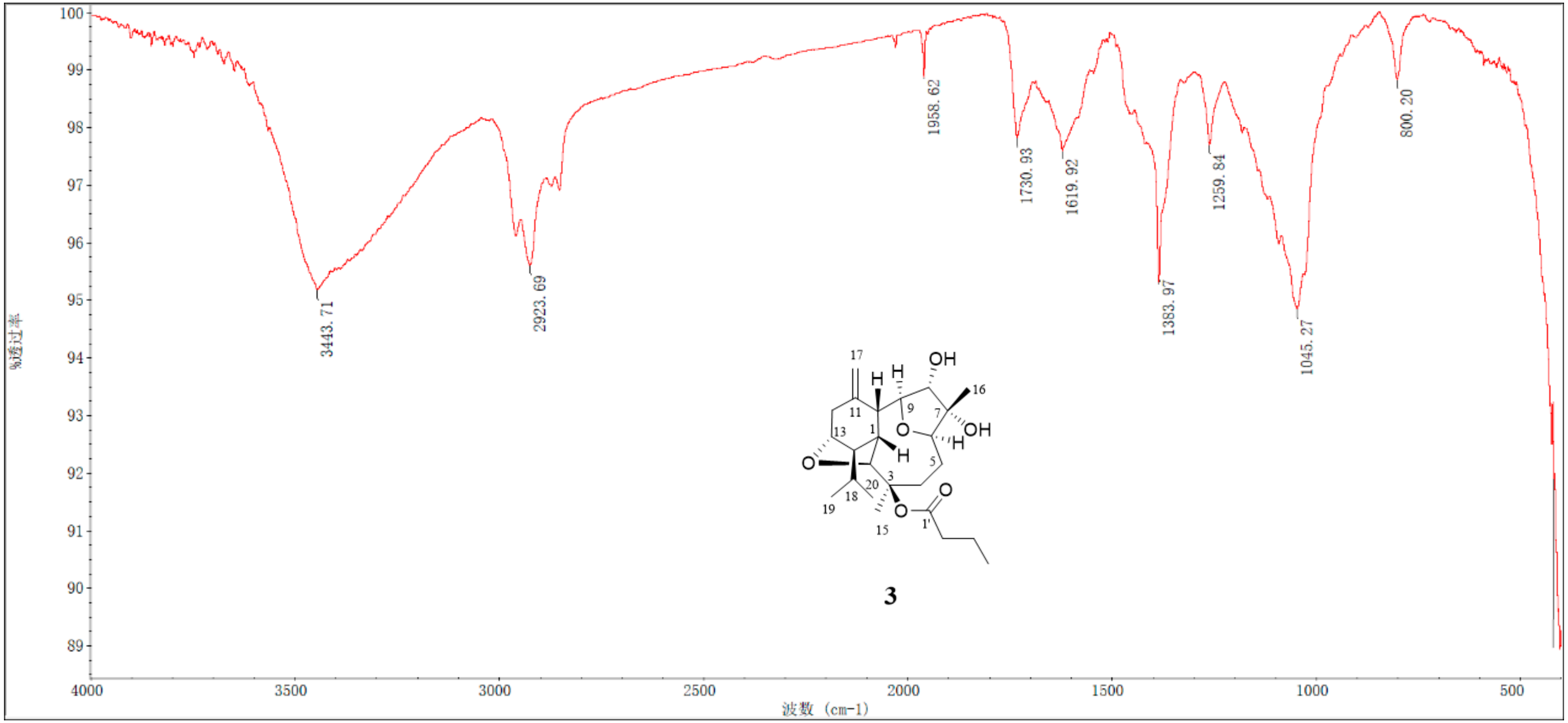

Figure S24. IR spectrum of ximaoornatin C (**3**).

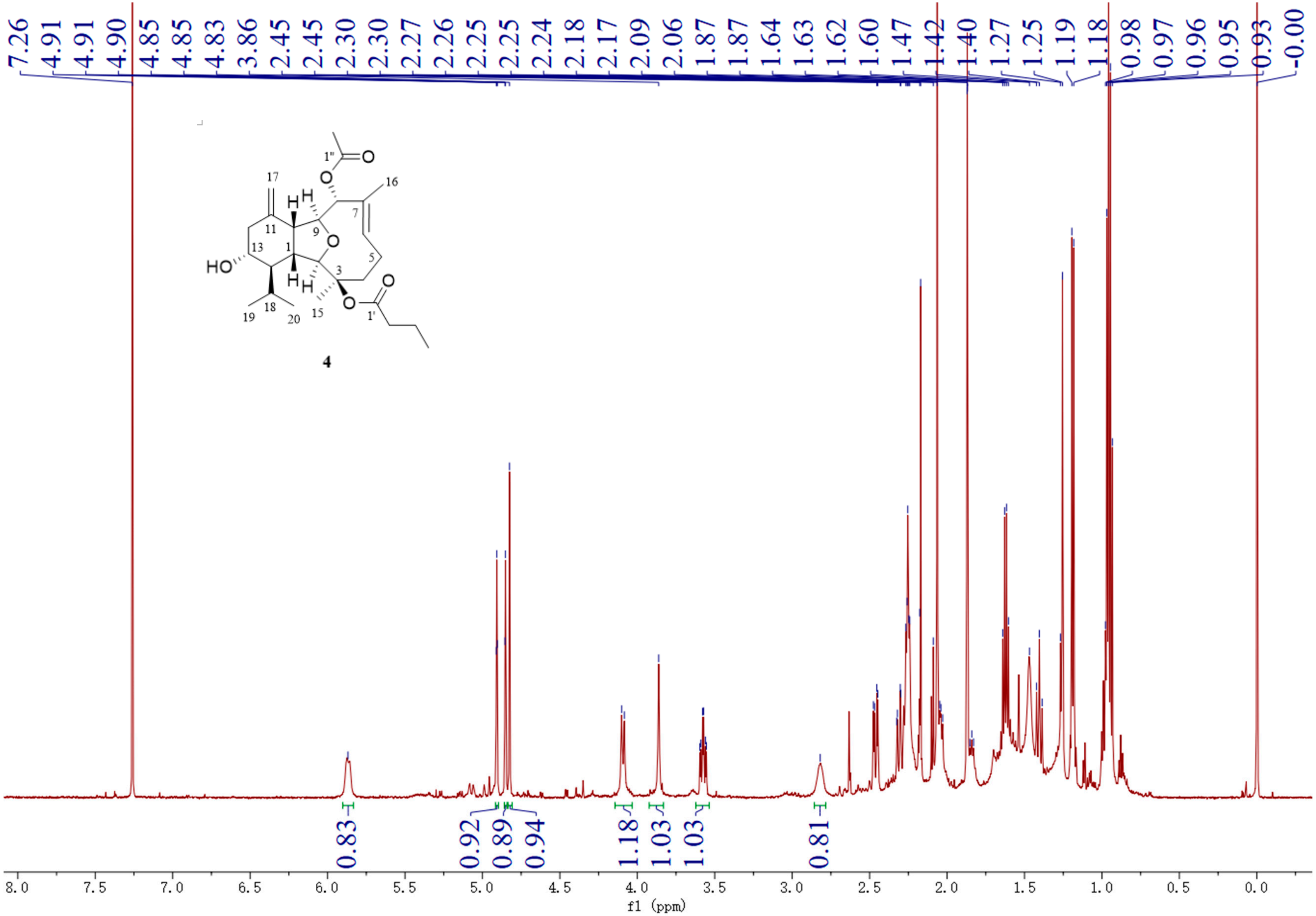

Figure S25. ^{1}H NMR spectrum of litophynin K (**4**) in $CDCl_3$.

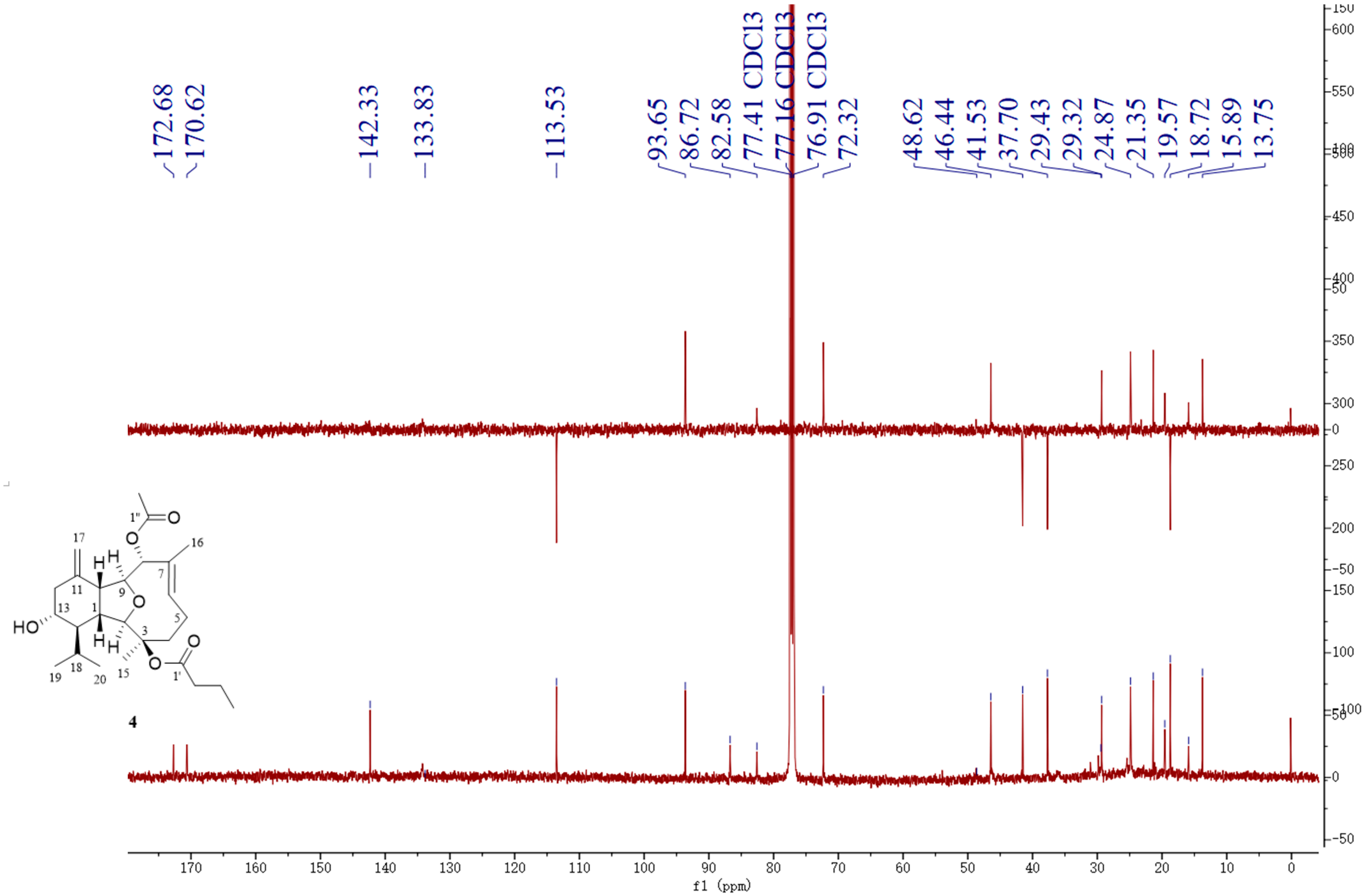

Figure S26. ^{13}C NMR spectrum of litophynin K (**4**) in $CDCl_3$.

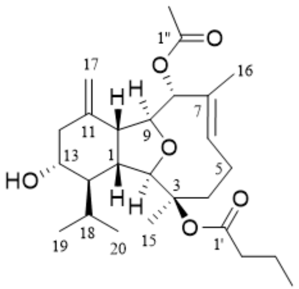

4

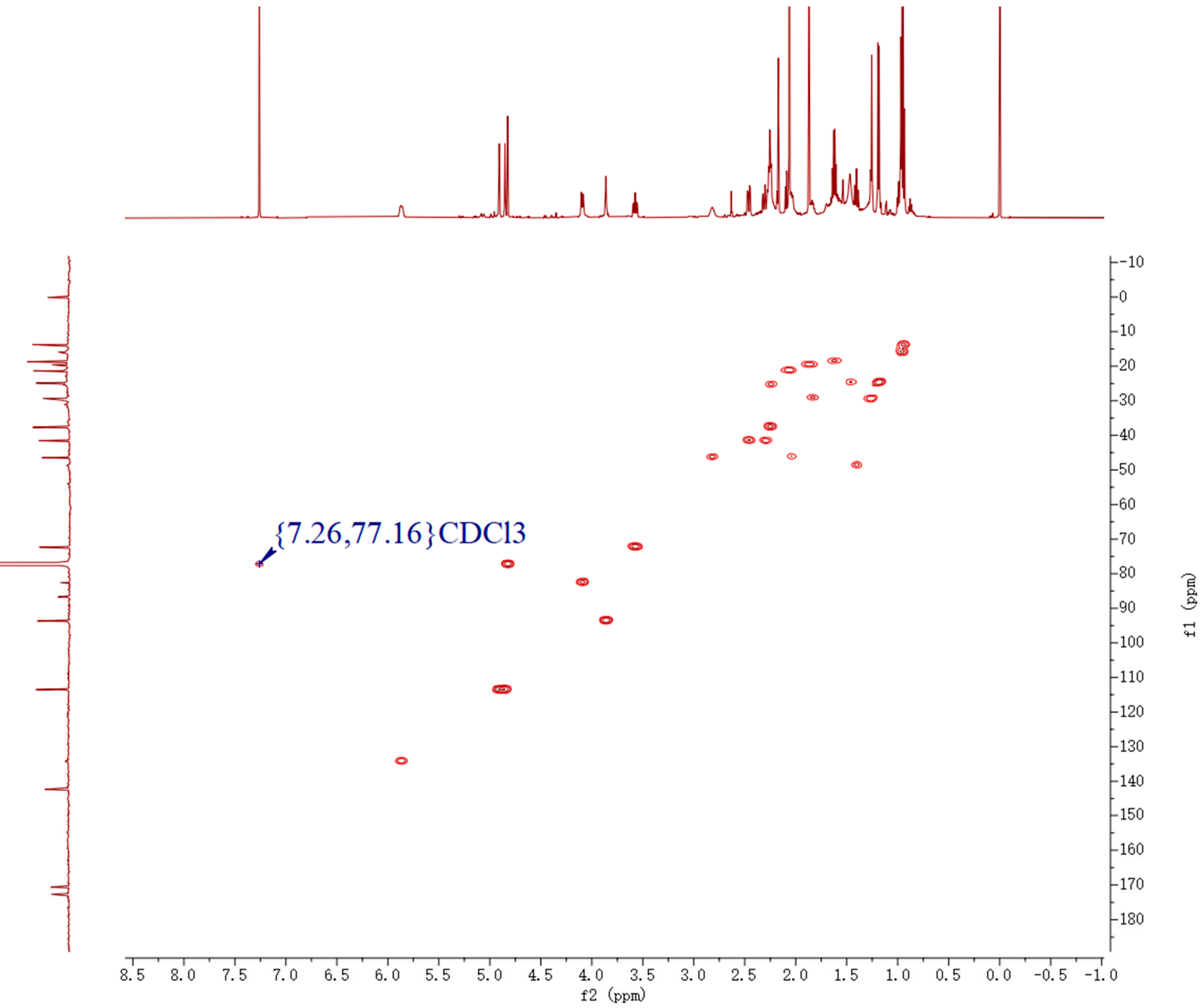

Figure S27. HSQC spectrum of litophynin K (**4**) in $CDCl_3$.

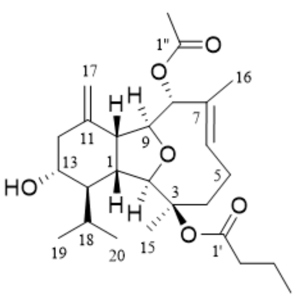

4

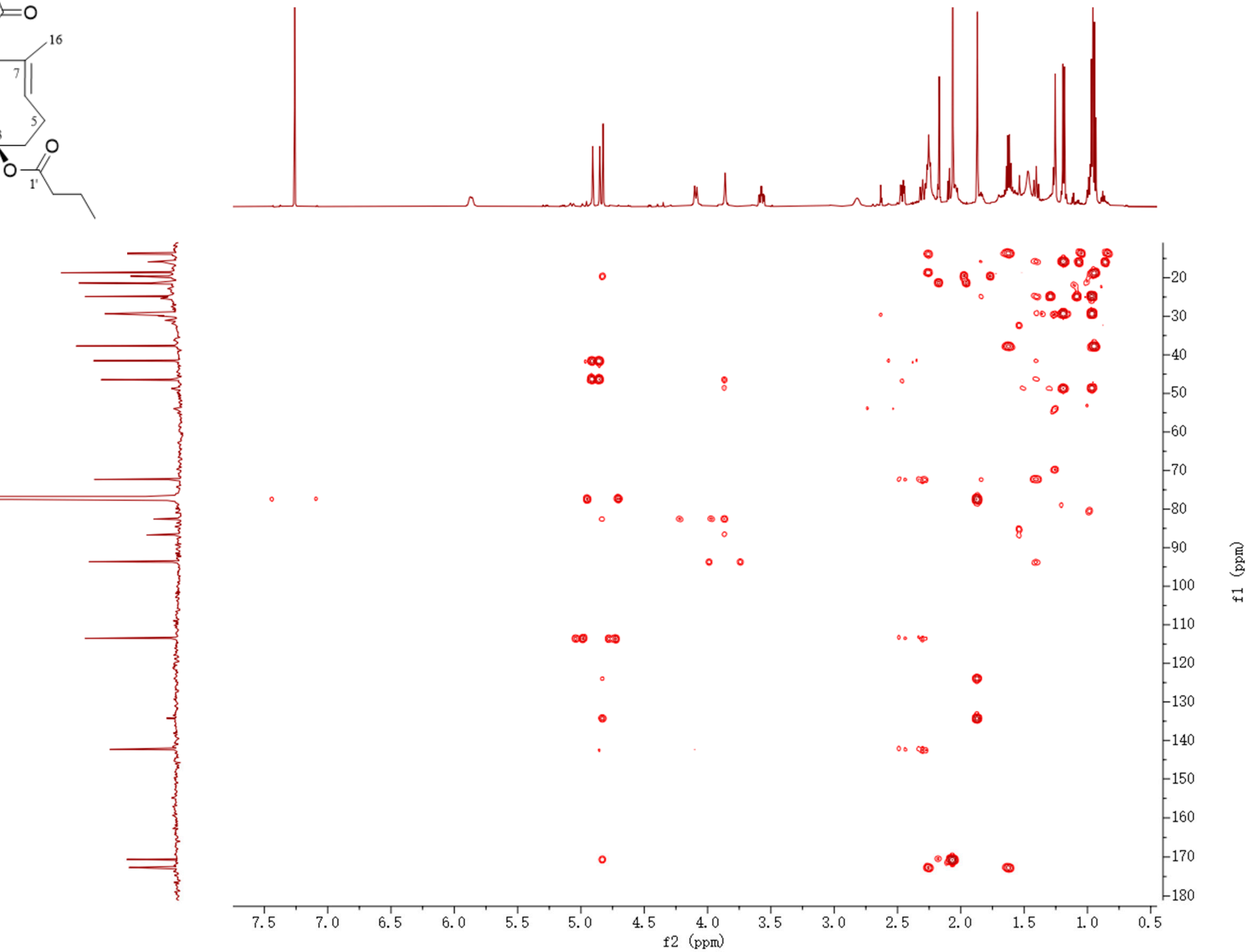

Figure S28. HMBC spectrum of litophynin K (**4**) in $CDCl_3$.

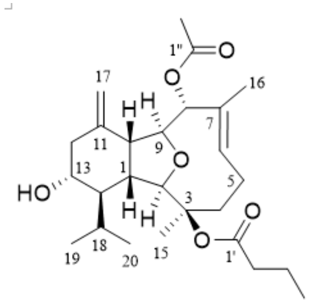

4

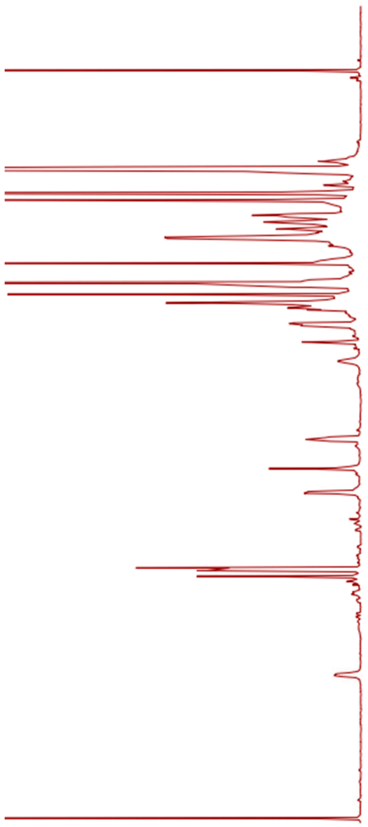
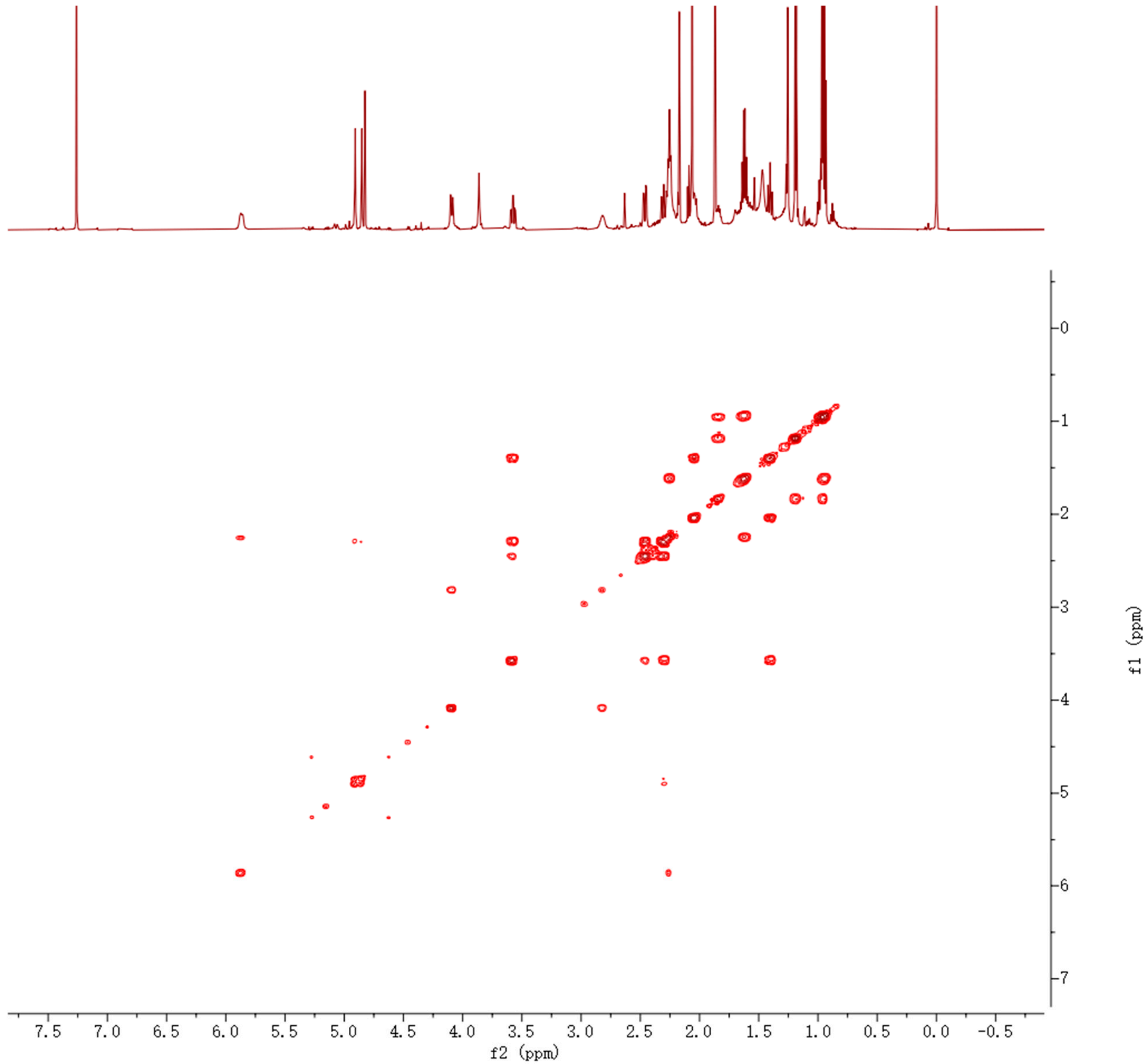

Figure S29. ^{1}H-^{1}H COSY spectrum of litophynin K (**4**) in $CDCl_3$.

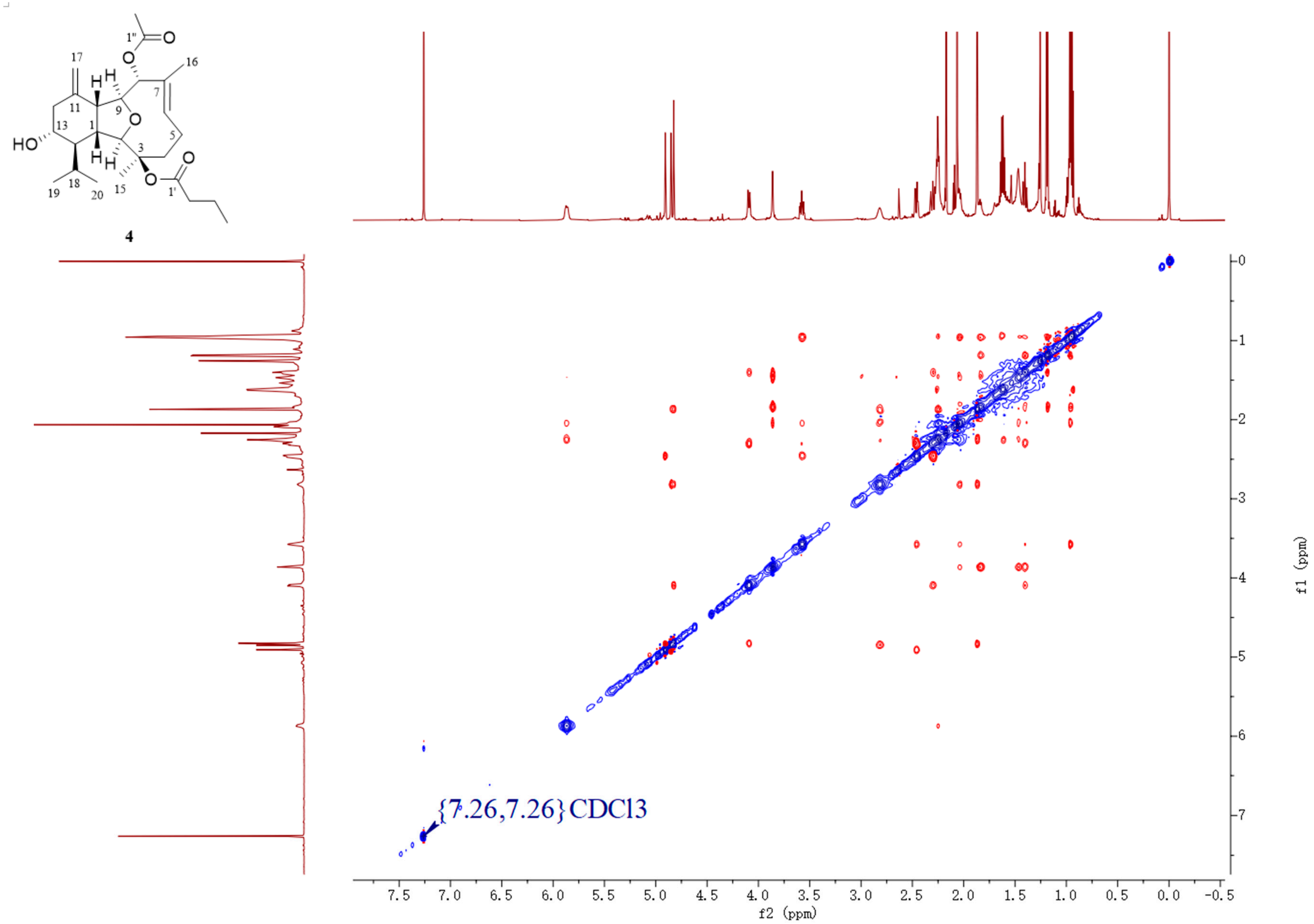

Figure S30. NOESY spectrum of litophynin K (**4**) in $CDCl_3$.

Qualitative Analysis Report

**Data Filename**	ESIH_20200508_GYW_SLL_07.d	**Sample Name**	A8-DBE-6
**Sample Type**	Sample	**Position**	P1-C7
**Instrument Name**	Agilent G6520 Q-TOF	**Acq Method**	20160322_MS_ESIH_POS_1min.m
**Acquired Time**	5/8/2020 16:31:08	**IRM Calibration Status**	Success
**DA Method**	small molecular data analysis method.m	**Comment**	ESIH by ZZY

User Spectra

Fragmentor Voltage	Collision Energy	Ionization Mode
135	0	ESI

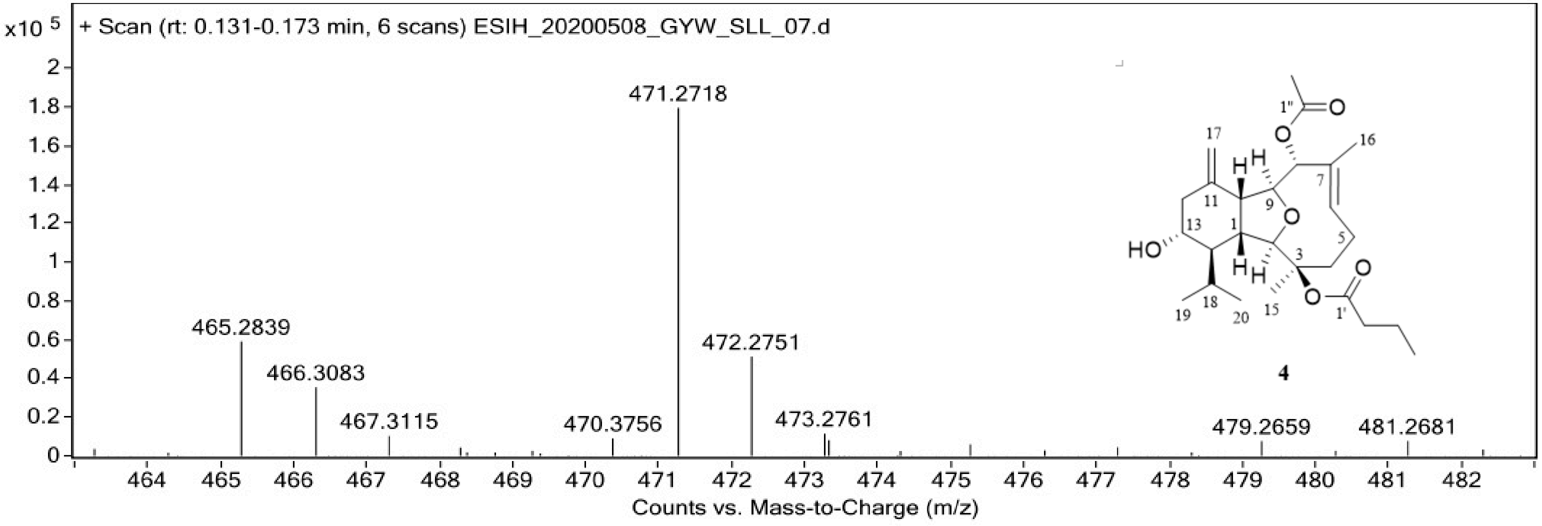

Formula Calculator Results

m/z	Calc m/z	Diff (mDa)	Diff (ppm)	Ion Formula	Ion
471.2718	471.2717	-0.08	-0.17	C26 H40 Na O6	(M+Na)+

--- End Of Report ---

Figure S31. HRESIMS spectrum of litophynin K (**4**) in MeOH.

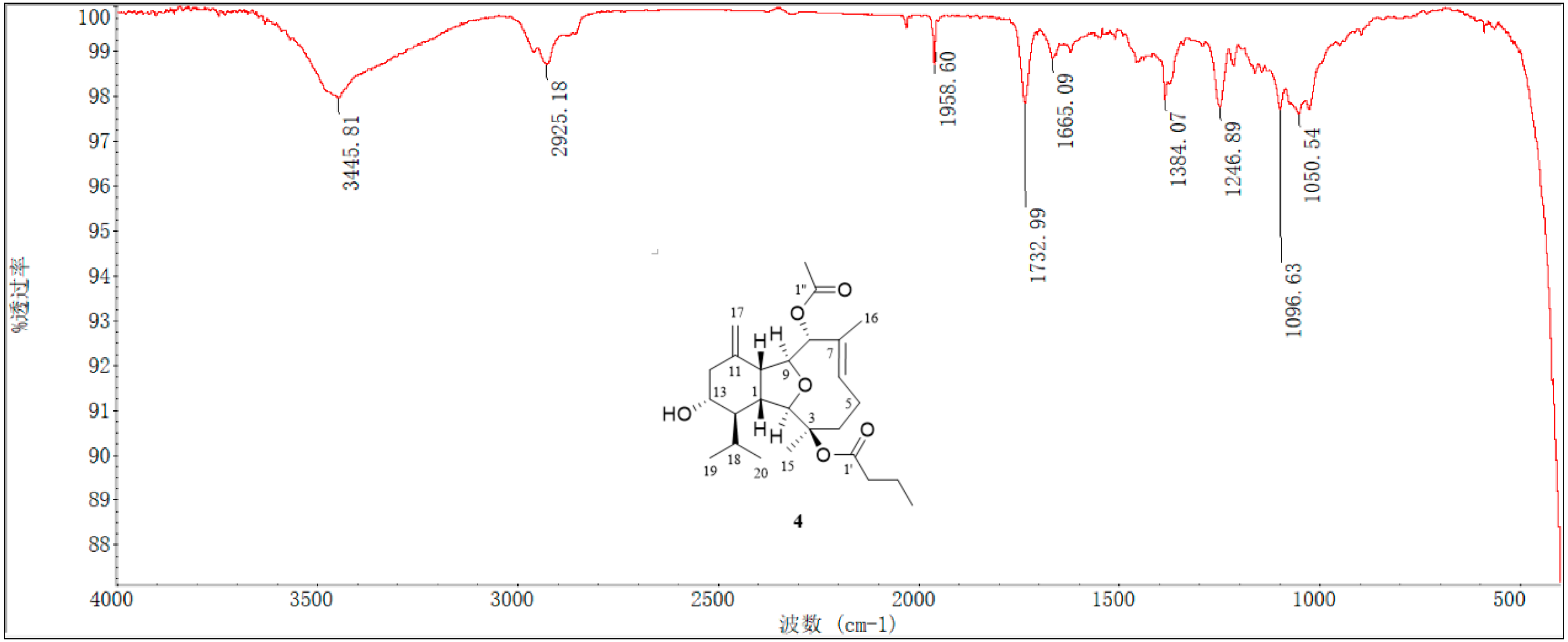

Figure S32. IR spectrum of litophynin K (**4**).

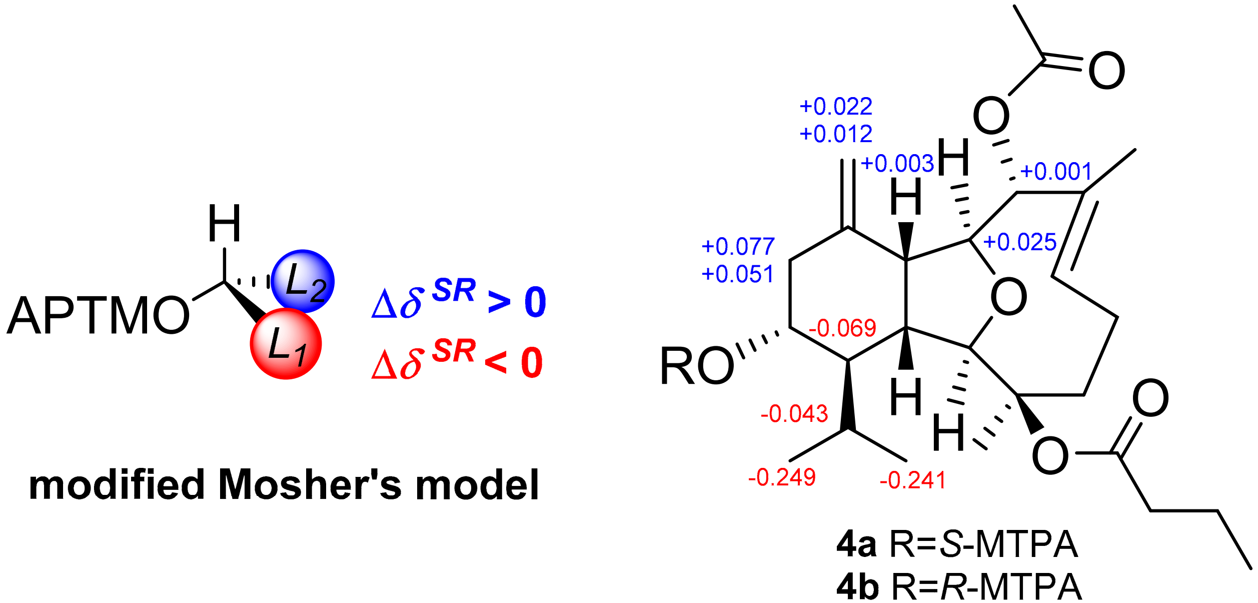

4a R=*S*-MTPA
4b R=*R*-MTPA

Figure S33. Application of the modified Mosher's method for AC determination of the secondary alcohol on **4**. Values of $\Delta\delta^{SR}$ [$\Delta(\delta_S - \delta_R)$] were given in ppm. Regions (+ and −) were marked in blue and red, respectively.

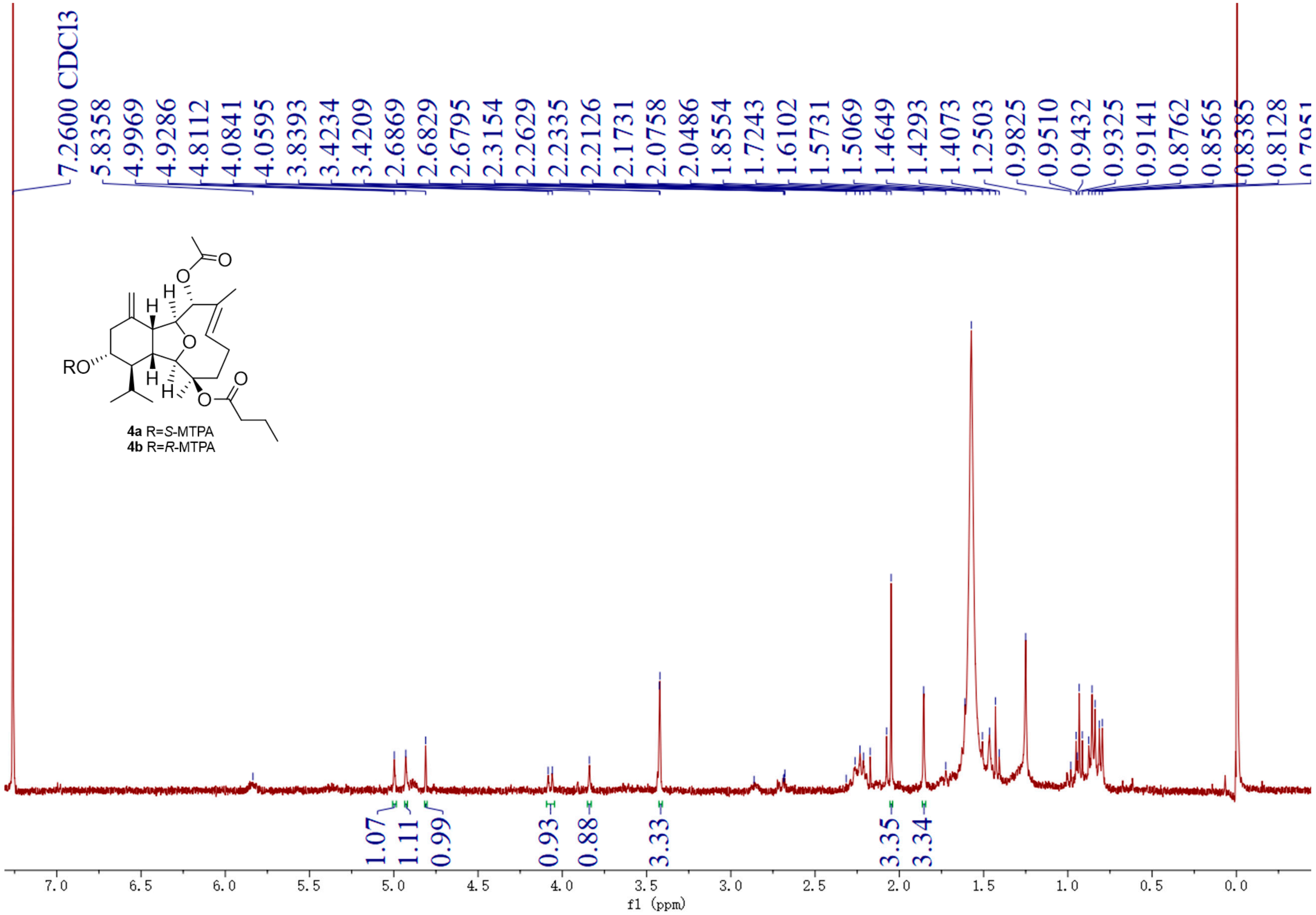

Figure S34. ^{1}H NMR spectrum of **4a** in $CDCl_3$.

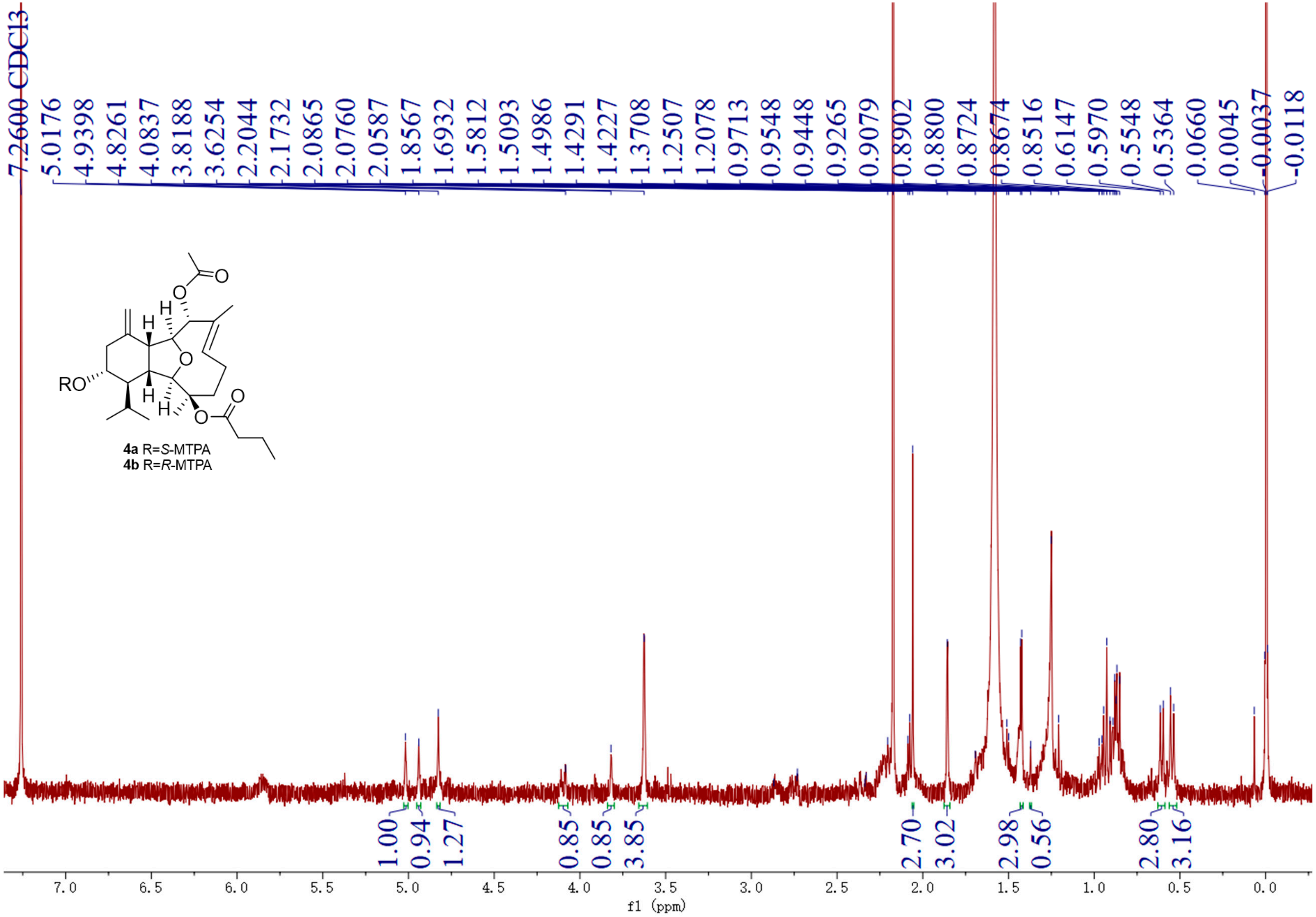

Figure S35. ^{1}H NMR spectrum of **4b** in $CDCl_3$.

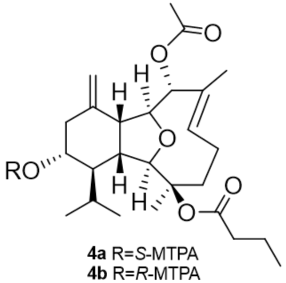

4a R=*S*-MTPA
4b R=*R*-MTPA

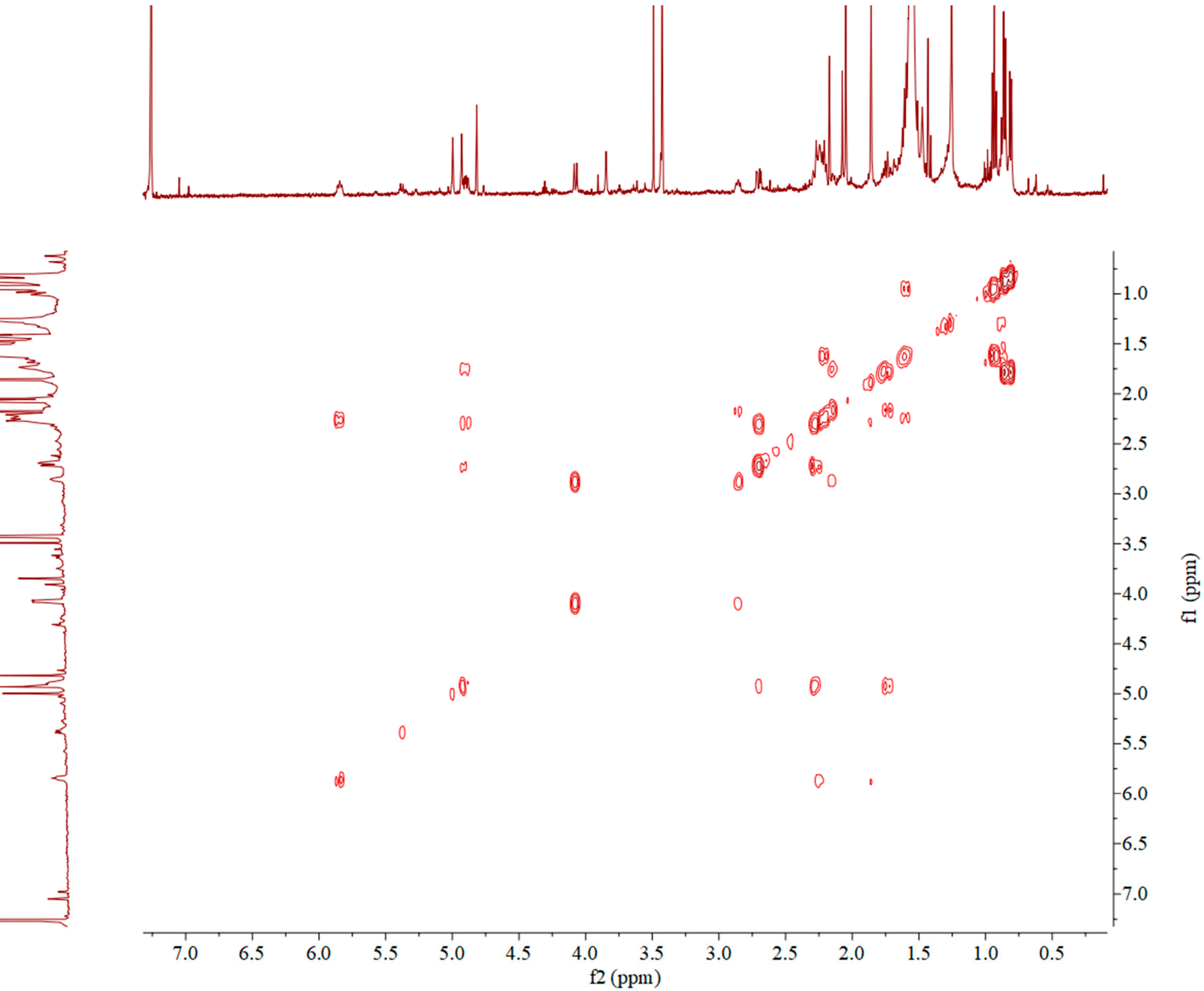

Figure S36. ^{1}H-^{1}H COSY spectrum of **4a** in CDCl$_3$.

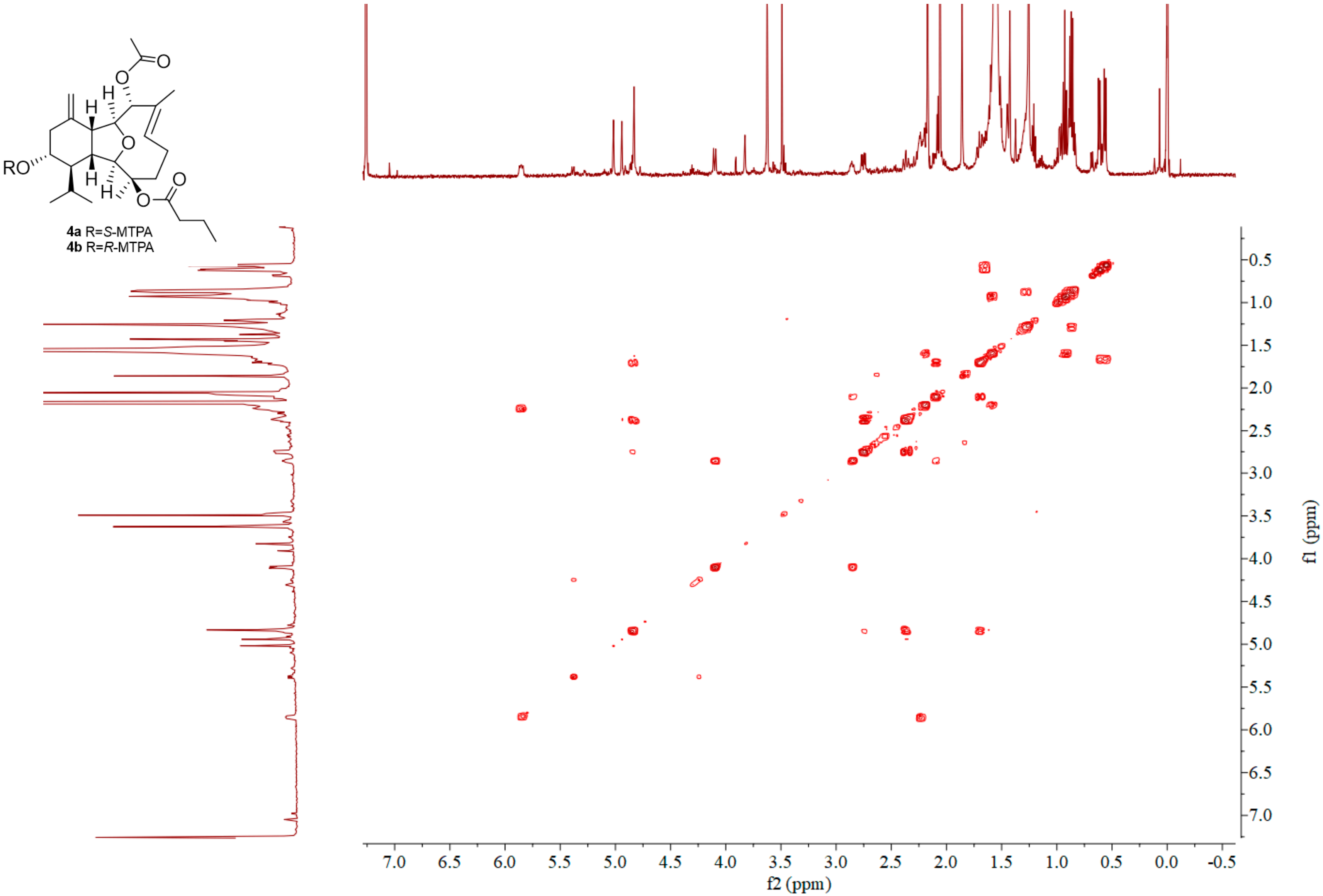

4a R=*S*-MTPA
4b R=*R*-MTPA

Figure S37. ^{1}H-^{1}H COSY spectrum of **4b** in $CDCl_3$

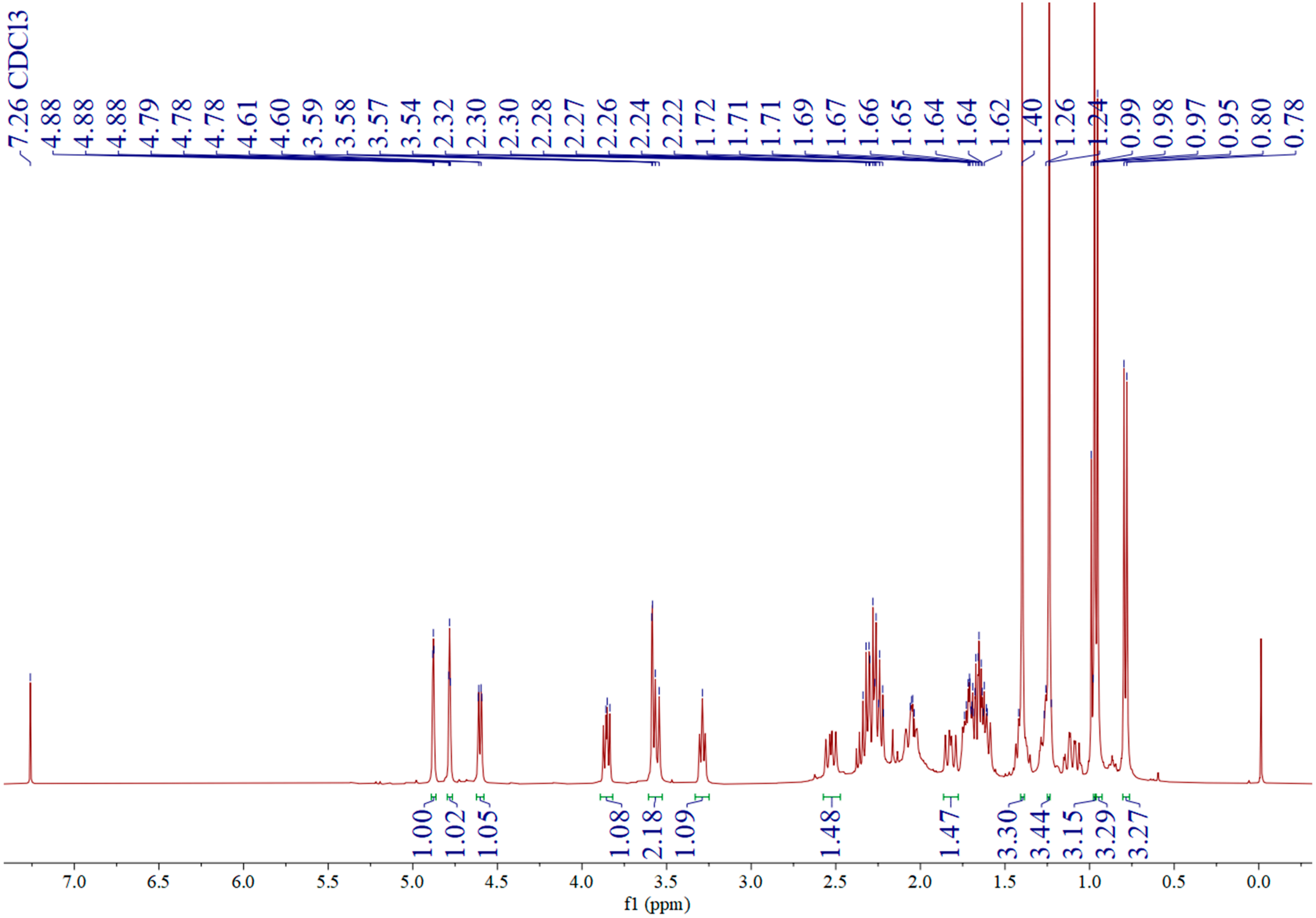

Figure S38. ^{1}H NMR spectrum of litophynol B (**5**) in $CHCl_3$.

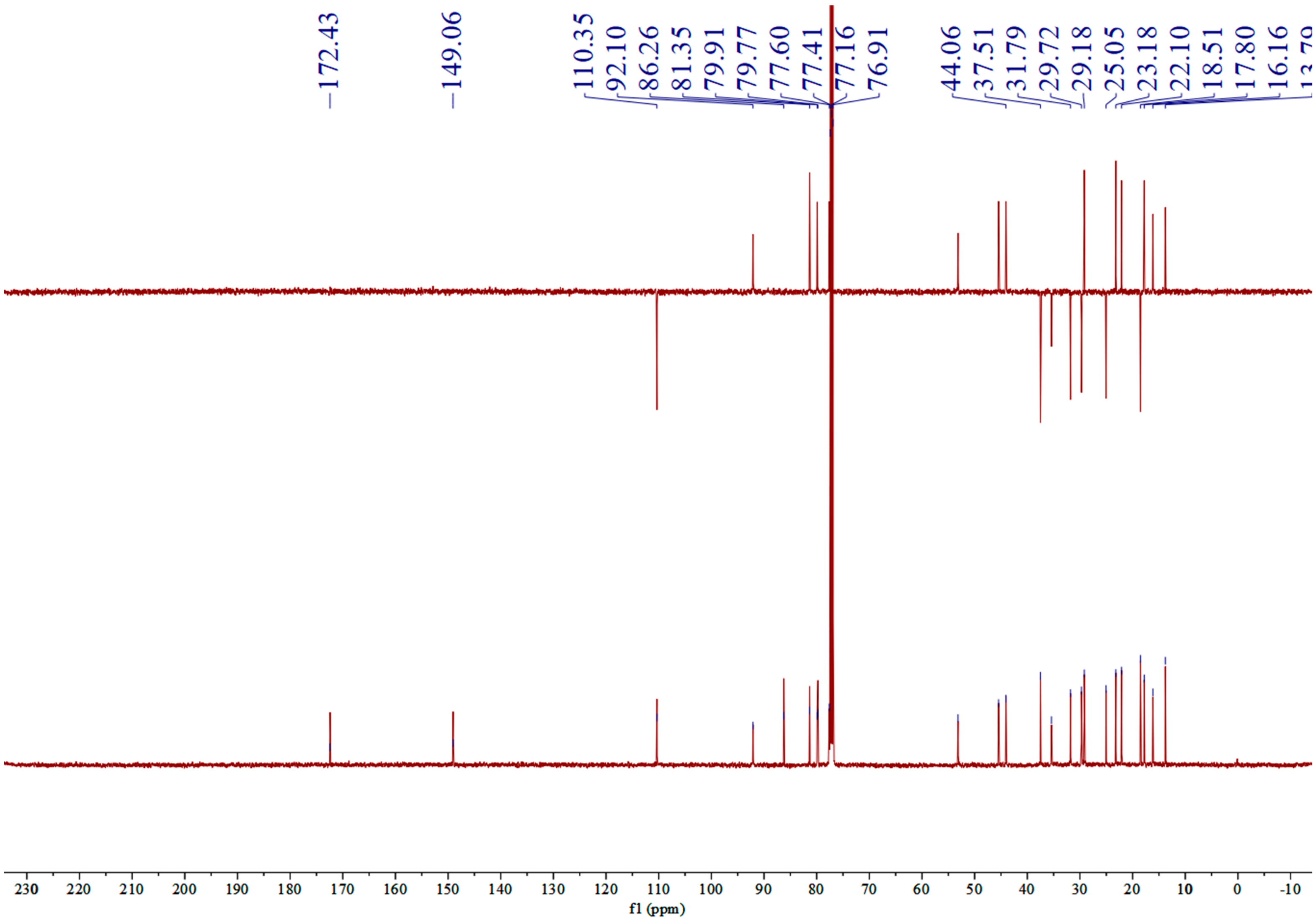

Figure S39. ^{13}C NMR spectrum of litophynol B (**5**) in CH
